# A Survey on the Energy Detection of OFDM Signals with Dynamic Threshold Adaptation: Open Issues and Future Challenges

**DOI:** 10.3390/s21093080

**Published:** 2021-04-28

**Authors:** Josip Lorincz, Ivana Ramljak, Dinko Begušić

**Affiliations:** 1 Faculty of Electrical Engineering, Mechanical Engineering and Naval Architecture (FESB), University of Split, 21000 Split, Croatia; dinko.begusic@fesb.hr; 2 Elektroprenos–Elektroprijenos BiH a.d. Banja Luka, 88000 Mostar, Bosnia and Herzegovina; ivana.marincic1988@gmail.com

**Keywords:** adaptive algorithms, cognitive radio, energy detection, OFDM modulation, detection probability, spectrum sensing, wireless networks, radio spectrum management, signal detection, dynamic scheduling

## Abstract

Cognitive radio (CR), as a concept based on the ability to detect and share the unutilised spectrum, has been envisioned as a promising candidate to improve the efficiency of frequency spectrum assignments. For the realisation of the CR concept, energy detection (ED), as one of the available spectrum sensing methods, is broadly considered because of its low computational complexity and implementation costs. Due to the vast usage of the orthogonal frequency division multiplexing (OFDM) technique in contemporary communication systems, the ED of OFDM signals in the CR networks has become important for practical realisation. Since the ED accuracy of the OFDM signals can be improved by the sensing threshold adaptation, this paper surveys the impact of noise variations and dynamic threshold (DT) adaptation on the ED performance of OFDM signals. Analyses were performed by the simulation of the ED related to OFDM signals transmitted in the margin or rate adaptive and combined margin and rate adaptive OFDM systems. The results obtained through extensive simulations provide fundamental insights into how different factors, including the transmission power, the signal to noise ratio, the false alarm probability and the sample quantity, affect the ED efficiency. Comprehensive analyses of the obtained results indicate the main ED weaknesses and how the appropriate selection of analysed factors can enhance the ED processes for different OFDM systems. The observed ED weaknesses were further thoroughly surveyed, and the open issues and challenges related to the enhancement of the main ED limitations have been elaborated. The presented survey results can serve as a basis for the improvement of a broadly accepted ED method in CR networks.

## 1. Introduction

Due to the rapid growth in the number of wireless devices, the exponential increase in the number of new applications and the continuous demand for higher data rates, the radio frequency (RF) spectrum has become increasingly crowded. In addition, RF congestion is further contributed to by the growing demand for mass spectrum access, particularly for social and personal applications. The problem of a congested RF spectrum becomes even more severe with the implementation of technologies like 5th generation (5G) cellular networks and the Internet of Things (IoT). These trends in communication networks request a new generation of devices that will be aware of their RF surroundings and which will facilitate efficient, flexible and reliable operations and the utilisation of available spectral resources. Analyses indicate that many unlicensed and licensed parts of the RF spectrum are not optimally exploited and that the idle and occupied periods of the RF spectrum vary in both time and space domains [1,2,3]. Cognitive radio (CR) as a concept based on the ability to detect and share the unutilised spectrum has been envisioned as a promising candidate for improving policy related to the inefficient assignment of frequency spectrum [4]. In CR, spectrum sensing (SS) is seen as the most demanding process. The goal of SS is to detect the periods of inactivity of the licensed user, known as the primary user (PU), in a specific frequency band and, if this band is available, to enable transmission for an unlicensed user, known as a secondary user (SU), such that it does not interfere with the PU. Thus, the utilisation of an unutilised band by the SU in the moments when the PU is not active can significantly reduce the spectrum scarcity problem [5,6].

Different approaches have been proposed in the literature for SS. Energy detection (ED) as one of the non-cooperative methods of SS is broadly considered due to its low computational complexity and simple implementation. The main advantage of the ED method is the fact that it does not include complex signal processing and does not require prior information about the PU signal. However, ED has some limitations, such as the need for a priori knowledge on the noise energy level or its reliable estimate, a susceptibility to the noise power uncertainty (also known as noise uncertainty (NU)), a poor performance below a certain value of Signal-to-Noise Ratio (SNR) level (known as noise floor level) and the lack of ability to distinguish between PU signals, SU signals and interference [3,7].

In essence, the ED technique includes an estimation of the received signal energy at the receiver side and a comparison of the estimated energy, with a set threshold to confirm the presence or absence of the PU signal. The SS performance of the ED method depends greatly on the setting of the detection threshold [8]. Setting an appropriate threshold is a challenging task since it must differentiate among the PU signal and noise. Dynamic threshold (DT) adaptation and the fixed threshold approach are two main approaches to set the detection threshold of the PU signal in the ED process.

A conventional energy detector uses a fixed threshold value to judge the occupation of the spectrum. In this case, regardless of the SNR fluctuations, the fixed threshold value does not change over time. The disadvantage of this approach is that the system requires prior knowledge of the noise level. The threshold is determined as the most appropriate and it is set manually as a static level above a noise floor. If the static threshold is set too high above the noise floor, the percentage of PUs that will remain undetected can increase, which may cause harmful interference from the SU to the PU. Hence, due to the fluctuating nature of the noise signals that exist in practice, this approach is susceptible to erroneous decision-making. Some fixed threshold techniques that have been proposed in the literature are histogram analysis, the empirical analysis of spectrum measurements, the receiver noise characteristics threshold and the P-tile-based threshold technique [9,10,11].

Unlike the fixed threshold technique, the dynamic (adaptive) threshold approach adjusts the threshold values to variations in the noise, enabling the SU to dynamically adapt its detection threshold according to the current SNR, sensing time or PU transmit (Tx) power [10,12]. In the case of the DT approach, a priori knowledge of the PU signal and noise floor level is not required. Some of the DT techniques are Otsu’s algorithm, Principal Component Analysis, Maximum Normal Fit and Recursive One-Sided Hypothesis Testing [9,10,12,13,14,15].

Although the DT approach is more demanding in terms of the practical implementation than the static threshold approach, stated references show that the DT approach improves the performance of the energy detector. This is a consequence of the fact that, in realistic conditions, thermal noise and interference from other remote communication systems causes noise variation (also known as NU). This NU in turn contributes to the variation in the SNR of the SU [16]. While NU in SS makes the detection process with a fixed threshold unreliable, by contrast, the DT technique can cope with NUs through DT adaptation. Hence the techniques based on DT are more robust to noise [9,17], which motivates the investigation of DT adaptations in relation to ED performance in this survey.

Additionally, in this work, the performance evaluation of the ED process based on DT was performed for the Orthogonal Frequency-Division Multiplexing (OFDM) signals received at the location of the SU. OFDM was selected for analysis since it is seen as a promising candidate for use in cognitive radio networks (CRN) due to the implementation of a cyclic prefix (CP) for mitigating multipath fading and reducing inter-symbol interference (ISI) [14,18,19]. OFDM has become the modulation of choice in a large number of wireless broadband systems, which explains the necessity of performing analyses on the ED performance for OFDM signals.

Versatile modulations are used in communication systems based on OFDM that includes 16/64/256/1024/2048… Quadrature Amplitude Modulation (QAM), Quadrature Phase-Shift Keying (QPSK) and Binary Phase Shift Keying (BPSK). The choice of the optimal modulation technique depends on the cost-effectiveness of the system, the ability to provide larger data rates, Bit Error Rate (BER) and SNR [20,21]. The OFDM modulation selection is closely related to the OFDM system design. In practice, three different approaches are used for OFDM systems, namely the margin adaptive (MA) dedicated to the minimisation of the transmit (Tx) power with respect to the BER and transmission rate constraints, the rate adaptive (RA) dedicated to the maximisation of the transmission rate with respect to the BER and Tx power constraints, and the combined MA and RA approach, which jointly optimises the Tx power and transmission rate under a BER constraint [22]. Since the ED process is impacted by each of these OFDM system design approaches, comprehensive analyses were performed in this work to perceive this impact.

Additionally, this paper surveyed the literature on the evolution of ED as one of the most represented methods for local SS in cognitive radio networks. The influence of DT adaptations on ED performance concerning the signals transmitted using distinct OFDM-based systems was also analysed. The analyses were performed using a developed simulation algorithm that enables simulation of the ED process. The proposed algorithm simulates ED of PU signals based on the DT adaptation for each OFDM system design impacted by different NUs. This survey includes a pseudocode of the simulation algorithm in order to show the practical aspects of the developed algorithm. Simulation is performed for versatile simulation scenarios, differing in the selection of impact of the NU level and DT adaptation on the ED of differently modulated OFDM signals.

The main contributions of this paper are:

- mathematical formulation and explanation of the SS models which take into account the impact of NU variations, DT adaptations, and both, on the probability of ED,
- presentation of the algorithm developed for simulating ED performance on differently modulated OFDM signals detected in MA, RA, and combined RA and MA based implementations,
- a systematic investigation based on extensive simulations on how the different OFDM modulations and corresponding Tx powers, the SNRs in the position of SU, the levels of NUs, the sample quantity, and the false alarm probability impact on the probability of signal detection in the ED process with DT adaptation,
- explanation of the limitations of ED as one of the local SS methods, with discussion on future research challenges and opportunities related to the improvement of the ED technique.

The rest of the paper is structured as follows. In Section 2, an overview of the topic related to the SS of OFDM signals is presented. Section 3 describes communication technologies and versatile communication systems, which use OFDM as a transmission technique. ED of OFDM signals as the SS model is introduced in Section 4. The mathematical formulation of SS models which take into account the impact of NU variations, DT adaptations and both on the probability of ED is presented in Section 5. The algorithm used for simulation of the ED process based on DT adaptations is presented in Section 6. The extensive simulation results to determine the ED of signals transmitted using distinct OFDM-based systems are presented and surveyed in Section 7. Based on the analyses presented in Section 7, the main ED drawbacks and future research challenges and opportunities for future research relating to the improvement of ED performance are thoroughly discussed in Section 8. Finally, some concluding remarks are given in Section 9.

## 2. Related Works on the SS of OFDM Signals

**Figure 1:**
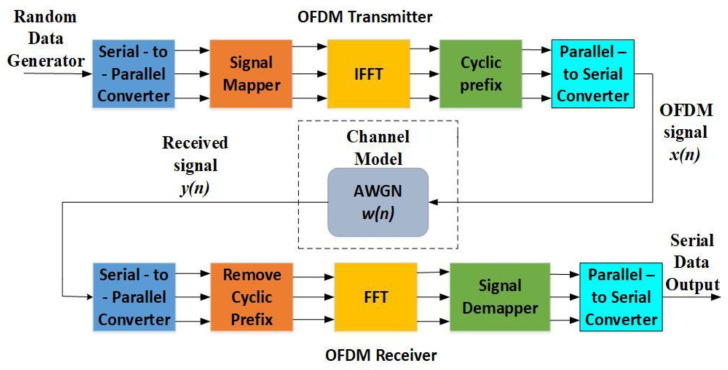
Block diagram of OFDM transmission and reception process.

OFDM is a multiplexing scheme based on the idea of transmitting data using a number of orthogonal subcarriers [20]. This orthogonality can reduce the interference between the subcarriers and increase spectrum efficiency utilisation. Figure 1 presents the block diagram of the OFDM system consisting of three main subsystems: a transmitter, wireless channel and receiver.

Firstly, randomly generated data is sent to the transmitter block serially. Then, a serial-to-parallel conversion is performed. To modulate the signal for transmission, one of the possible modulation schemes (e.g., BPSK, QPSK, 16/64/256/… QAM) is used. The parallel bits of data are mapped to the subcarriers using an Inverse Fast Fourier Transform (IFFT) block. During the further signal processing, a cyclic prefix (CP) is inserted into the OFDM symbol and a guard interval (GI) is appended to each block of data to combat inter-symbol-interference (ISI). After the insertion of the CP and GI, the resultant OFDM symbols are converted to serial form and transmitted through a channel. To extract the original information, the OFDM receiver performs processes that are the reverse of those performed by the OFDM transmitter (Figure 1) [23].

The SS of OFDM signals transmitted according to the described transmission process has been analysed in different studies in the literature [10,11,19,24,25,26,27,28,29,30,31,32,33,34,35]. In Table 1, an overview of ED methods and their corresponding merits are presented. In Ref. [24], the relationship between the probability of signal detection and the probability of assuming the existence of PU when PU is not actually present (also known as a false alarm probability) was analysed. The analyses were performed for the ED of BPSK signals transmitted in Wireless Local Area Network (WLAN) and Worldwide Interoperability for Microwave Access (WiMAX) systems. It is shown that a major challenge for the ED SS is the inability to sense OFDM signals at low SNRs [10,25].

The SS using ED of OFDM signals transmitted with QPSK modulation is proposed in Ref. [11] (Table 1). To estimate the sensing threshold, an adaptive inverse cumulative density function method was used. The disadvantages of the simulated ED method were analysed by defining how the detection capabilities of an implemented real ED corresponds to the simulated equivalent. In Ref. [41], the detection of narrowband OFDM signal transmission by means of ED method was proven to be feasible, with the possibility of extending SS based on the ED method to more complex OFDM systems. It is also shown that in a system where NU exists, even if the threshold is set adaptively, the presence of any in-band interference can reduce the detection precision of the energy detector.

In Refs. [26,27], based on the CP feature of the OFDM signal, the Neyman-Pearson SS approach for OFDM signal detection was presented. This showed the extreme sensitivity of the proposed SS method to NU. Additionally, in Refs. [19,28,29], algorithms considering properties of the OFDM CP for SS were presented. The expression for selecting the sensing threshold is derived from Ref. [28] and has proven to be accurate and robust when put through simulations (Table 1). The proposed detection is capable of identifying weak OFDM signals due to its high sensitivity. The ED algorithms proposed in Refs. [19,29] proved to be sensitive to the changes in timing offset. In Ref. [27], a generalised ratio test based on log-likelihood in which the false alarms and signal detection probability are independent of the timing offset were studied.

A tutorial on the ED method and the thorough analysis of the test statistic in the ED process, without addressing the NU problem and DT adaptation, was given in Ref. [30]. Ref. [31] proposed a new technique for the SS of OFDM signals impacted with NU (Table 1). The results of simulations indicated that the proposed detector based on the mean ambiguity function can yield good performance of energy detection in environments with low SNR and can be persistent to NU.

Recent research attempts have been dedicated to finding the optimal detection threshold for SS based on ED (Table 1) [37,38,39,40]. In Ref. [38], an approach based on a double threshold for an unequal scale sampling was proposed to reduce the impact of NU and asynchronous primary user occurrence. The results of the simulation confirmed the effectiveness of the proposed approach in terms of improving the detection probability, while keeping the false alarm probability within the demanded range. The performance of an ED under NU, while employing an optimal threshold and DT correction with Chi-square and Gaussian distributions of the received signal power, was studied in Ref. [39]. The results obtained showed that the selection of an optimal threshold in the ED process impacted by NU reduces the probability of error. A novel approach to DT selection based on an online learning algorithm was proposed to improve the performance of ED and the matched filter method through minimization of the overall error probability [40]. Presented simulation results confirmed that optimal threshold selection improves the performance of SS. A three-event ED algorithm based on optimal DT adjustment was proposed in Ref. [37]. The proposed algorithm minimizes in one iteration the probability of ED error based on Newton’s method with forced convergence [37]. The developed method was analysed through simulations and results showed that the proposed method outperformed the conventional ED method. Although results presented in recently published literature showed that selection of optimal threshold improves ED performance (Table 1), they also showed that no universal approach to a dynamic selection of optimal threshold currently exists and that finding the optimal threshold represents a challenging task.

In Ref. [32], we showed the results related to the influence of NU on the energy misdetection probability of OFDM based systems in an MA system design. In addition, in Ref. [36] (Table 1), the analyses of the impact of NU on ED performed without DT adaptations for diverse OFDM based systems were shown. The results obtained indicated that the ED performance in different OFDM systems was significantly impaired by the NU. The results presented in this work are used as bases for analyses on how dynamic threshold adaptation impacts ED performance in OFDM systems which operate under versatile NU levels.

Previous research has shown that DT adaptation and NU variation have an impact on the ED process (Table 1). However, based on knowledge obtained through extensive analysis of previous works, the systematic and comprehensive presentation of results and cognitions related to the ED of OFDM signals are missing. This paper fills this gap, since it offers comprehensive analyses of the impact of NU and DT adaptation on ED performance of signals transmitted in OFDM systems using RA, combined MA and RA, and MA transmission schemes. Hence, in this survey paper, the computer simulation of the developed ED algorithm, presented with pseudocode, was used to overview the influence of DT adaptation and NU variation on ED effectiveness for distinct OFDM based systems. Furthermore, the main limitations of the ED method are thoroughly reviewed, and areas of new research and associated challenges related to the improvement of ED performance are discussed. Hence, the presented comprehensive survey related to the performance of the ED of OFDM signals can serve as a reference for the improvement of the ED as a widely used SS approach.

## 3. Design of Different OFDM Based Systems

**Figure 2:**
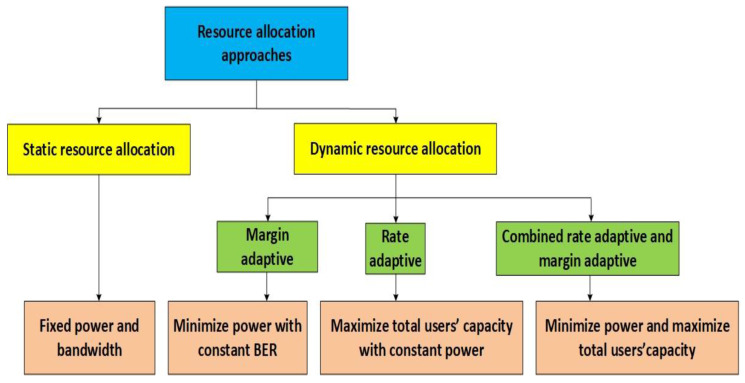
Different approaches to dynamic resource allocation in OFDM systems.

OFDM systems use three different types of design option (algorithm) for signal transmission (Figure 2). The first algorithm is based on the rate-adaptive (RA) design, which tends to maximise the instantaneous data rate with respect to the BER ad Tx power limitations. When the Tx power is kept at a fixed value in order to ensure equal BER (i.e., equal QoS), the transmitter must adjust the OFDM modulation scheme (m-PSK/m-QAM) according to the conditions in the wireless channel. A lower transmission rate realized through the selection of the lower constellation order (m) of the OFDM modulation will be used in the case of poor channel quality and vice versa. Different real OFDM systems transmit with fixed Tx power, since the concept which exploits adaptive modulation selection is simpler in terms of the practical implementation of circuit design [22]. Implementations of such OFDM systems include WLAN, WiMAX, etc. However, in RA systems, information about the quality of the wireless channel, commonly detected at the receiver end, must be returned through a reverse channel to the transmitter which will adjust the appropriate modulation.

The second OFDM design algorithm is based on margin-adaptive (MA) system design, which minimises the Tx power based on the BER and data rate limitations (Figure 2). To ensure the same QoS (i.e., the same BER) while keeping the unchanged transmission rate (constellation order m must be kept unchanged), the Tx power should be changed based on the conditions in the wireless channel: a higher Tx power where the quality of the wireless channel is poor, and vice versa [22].

The third algorithm takes into account the recently adopted power and bit loading design option, which functions as a combined MA and RA approach (Figure 2). This approach tends to minimise the Tx power and maximise the data rate with respect to the instantaneous BER constraints in the wireless channel [22]. The incentive to employ, in conjunction, margin and rate optimisation can be found in the newest wireless systems that operate in diverse conditions and requirements. More precisely, minimisation of Tx power is important when operating in shared interference-prone spectrum environments or near frequency-adjacent users. Additionally, if sufficient OFDM guard bands exist to differentiate the users, maximisation of throughput can be performed for the purpose of better utilisation of the wireless channel.

Besides OFDM-based transmission where users are only scheduled on the time domain scale, in the OFDM access (OFDMA) systems the users are scheduled on a frequency and time domain scale. Hence, OFDMA systems are based on OFDM and have a broad practical implementation, which is defined in IEEE 802.16 (WiMAX), IEEE 802.11ax (WLAN), IEEE 802.20 (mobile broadband wireless access), Log Term Evolution/Advanced (LTE/LTE-A) and 5G systems [6,42,43]. OFDMA is also a possible access method for the Wireless Regional Area Networks (WRAN) defined in the IEEE 802.22 standard.

## 4. Energy Detection Model

SS, as the most demanding activity of CR, enables SU (unlicensed users) to adapt to the environment by detecting spectrum portions unused by licensed network users (PU). The problem of SS can be mathematically expressed as a binary hypothesis [8,17,44]:

(1) $$\begin{array}{c} H_{0}:y_{i}\left(n\right)=w_{i}\left(n\right),\\ i=1,\ldots ,\;M,\;\;n=1,\;\ldots ,\;N\;\;\mathrm{if}\;\mathrm{PU}\;\mathrm{is}\;\mathrm{absent}\\ H_{1}:y_{i}\left(n\right)=x\left(n\right)+w_{i}\left(n\right),\\ i=1,\ldots ,\;M,\;\;n=1,\;\ldots ,\;N\;\;\mathrm{if}\;\mathrm{PU}\;\mathrm{is}\;\mathrm{present} \end{array}$$

where $w_{i}$(*n)* is the noise signal received by the *i-th* SU, $y_{i}\left(n\right)$ is the signal received by the *i-th* SU during the *n-th* sample, x(n) is a transmitted signal from *i-th* PU impacted by the temporary amplitude gain of the channel at the point of discrete time sample *n*, and *N* is the overall sample quantity (number of samples) used in the sensing.

Period (number of samples) and *M* is the overall number of SUs [24,45,46]. In Table 2, all descriptions of parameters used in this work are presented.

**Figure 3:**
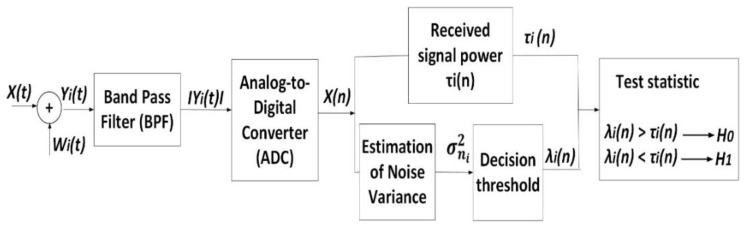
Block diagram of the energy detection process.

In Figure 3, a general block diagram of the detector based on the ED method is shown. The received signal $y_{i}$(t) consists of the signal of PU x(t) and the noise signal $w_{i}$(*t*). In the ED process, the received signal is passed through a band-pass filter (BPF) in order to select the signal bandwidth of interest and to remove out-of-band noises [24]. An analogue to digital converter (ADC) transforms the received signal in the digital domain and feeds it into two sub-processes (Figure 3).

In the first sub-process, the SU (cognitive radio) performs sampling and digital signal processing (Figure 3). The received signal is estimated via the magnitude square of the fast Fourier transform (FFT) and the result of this sub-process is the energy test statistic $\tau_{i}\left(n\right)$ obtained for an average of *N* samples, expressed as [45,47]:

(2) $$\;\;\tau_{i}\left(n\right)=\frac{1}{N}\overset{N}{\underset{n=1}{\sum }}{\left|y_{i}\left(n\right)\right|}^{2}$$

In the second parallel sub-process (Figure 3), the noise variance is evaluated according to SNR variations. Since modulation schemes like OFDM are designed to employ frequency diversity to provide robustness against fading, in this analysis, frequency-selectivity of multipath channels have been neglected. This is due to OFDM spreading of the wideband signal into many modulated narrowband subcarriers, which results in exposing those subcarriers to flat fading. Hence, the impact of noise received at the location of SU has been modelled in this analysis as additive white Gaussian noise (AWGN). AWGN is assumed to be an independent and identically distributed random process of zero mean and variance, $\sigma_{n_{i}}^{2}$, with a power spectral density, $w_{i}\left(n\right)\;\sim N$(0,$\sigma_{n_{i}}^{2})$.

The total variance of the received signal ($\sigma_{y_{i}}^{2})$ can be expressed as:

(3) $$\;\sigma_{y_{i}}^{2}=\sigma_{x}^{2}+\sigma_{n_{i}}^{2}=\sigma_{n_{i}}^{2}\left(1+SNR\right)$$

where $\sigma_{x}^{2}$ represents the variance of transmitted signal from the PU which can be expressed as $\sigma_{x}^{2}=SNR\;\sigma_{n_{i}}^{2}$. Assuming that there is no deterministic knowledge about the signal *x*(*n*) besides the average received power of the PU signal (which is characteristic of ED as a SS method), the average received power is expressed as:

(4) $$P=\frac{1}{N}\overset{N}{\underset{n=1}{\sum }}{\left|x\left(n\right)\right|}^{2}\approx \sigma_{x_{i}}^{2}$$

and the relation (3) can be transformed to $\sigma_{y_{i}}^{2}=P+\sigma_{n_{i}}^{2}$.

In the case of the known noise variance $\sigma_{n_{i}}^{2}$ and no uncertainty in noise variance (NU), $\tau_{i}\left(n\right)$ can be approximated using Gaussian distribution alongside a central limit theorem, where the hypotheses *H_o_* and *H_1_* (from relation (1)) become [45]:

(5) $$\tau_{i}\left(n\right)\;|\;H_{0}\sim N\left(\sigma_{n_{i}}^{2},\;\frac{2}{N}\sigma_{n_{i}}^{4}\right)$$

(6) $$\tau_{i}\left(n\right)|\;H_{1}\sim N\left(P+\sigma_{n_{i}}^{2},\frac{2}{N}{\left(P+\sigma_{n_{i}}^{2}\right)}^{2}\right)$$

To assess the performance of ED, a signal level that will be used as a decision threshold *(*$\lambda_{i}$*)* must be defined and comparison with the energy test statistic signal level ($\tau_{i}$) generated at the location of *i*-th SU must be performed. The decision threshold is determined based on the noise variance information obtained from the previous block, as in (Figure 3) [11,45,48]. The detection rule is defined by:

(7) $$\tau_{i}\left(n\right)>\lambda_{i}\left(n\right),\mathrm{PU}\;\mathrm{present}$$

(8) $$\tau_{i}\left(n\right)<\lambda_{i}\left(n\right),\;\mathrm{PU}\;\mathrm{absent}$$

where the signal level ($\tau_{i}\left(n\right)$) of the test statistic is obtained by calculating the received signal energy according to (2). This is obtained for a set of *n* samples and compared with the threshold $\lambda_{i}\left(n\right)$, which can be dynamically adapted and thus differs at the moment when a number of samples *n* is used for ED. Basically, the performance of the ED as an SS technique is done by examining the Neyman-Pearson hypothesis. To assess the Neyman-Pearson hypothesis, the comparison between the decision threshold and log-likelihood ratio of the received signal will be [11]:

(9) $$H_{0}:\;log\left(\frac{P(y_{0},\;y_{1,\;\ldots ,\;}y_{\left(N-1\right)}|H_{1})}{P(y_{0},\;y_{1,\ldots ,\;}y_{\left(N-1\right)}|H_{0})}\right)<\lambda_{i},\;\mathrm{PU}\;\mathrm{absent}$$

(10) $$H_{1}:\;log\left(\frac{P(y_{0},\;y_{1,\;\ldots ,\;}y_{\left(N-1\right)}|H_{1})}{P(y_{0},\;y_{1,\;\ldots ,\;}y_{\left(N-1\right)}|H_{0})}\right)>\lambda_{i},\;\mathrm{PU}\;\mathrm{present}$$

where $P(y|H_{0})$ and $P(y|H_{1})$ indicate the probability density functions (PDF) of the hypothesis $H_{1}$ and alternative null hypothesis $H_{0}$, respectively.

The hypothesis $H_{0}$ in relation (9) is valid if the received signal energy is lower than the set threshold ($\lambda_{i}$), and this confirms the existence of a spectrum hole. Based on the relation (10), another hypothesis $H_{1}$ is confirmed if the received signal energy is larger than the threshold value ($\lambda_{i}$), which leads to the cognition that the PU actively transmits [11,24,45]. The threshold $\lambda_{i}$ can be fixed or dynamically adjusted where threshold levels can be selected from the pool of values in a specific range, which is derived and explained in Section 5.

### 4.1. Receiver Operating Characteristic Curves

In order to evaluate the performance of SS techniques, several metrics are used. The most common are detection probability and false alarm probability. The probability that the SU correctly declares that a PU is present when the PU is really present is defined as the detection probability ($P_{d_{i}}$). The probability that SU incorrectly declares that the PU is present when the PU is actually absent is the false alarm probability ($P_{fa_{i}}$) [11].

**Figure 4:**
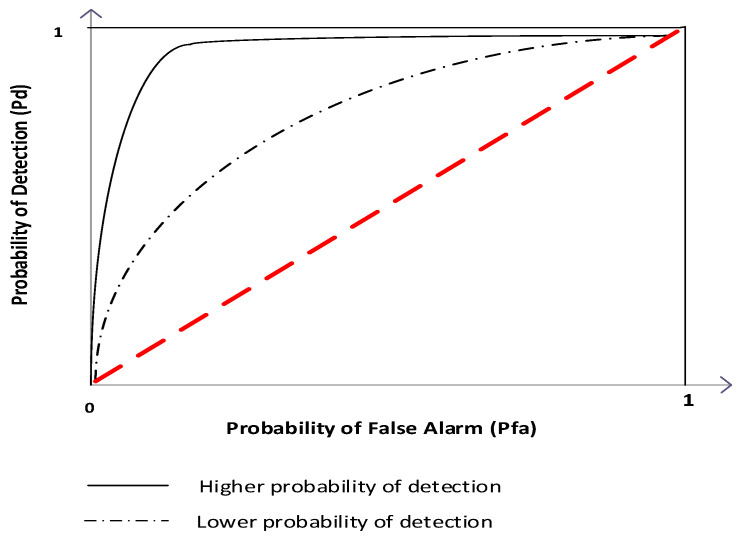
Example of ROC curves.

The interdependence between the probability of signal detection and the false alarm probability has been mostly expressed through receiver operating characteristic (ROC) curves (Figure 4). In Figure 4, the different spaces above and below the diagonal (also known as the line of no-discrimination) identify the quality of ED. If the ED process can be expressed as the line of no discrimination, this means that the quality of the ED process corresponds to a random guess. Generally, the ROC space above the diagonal represents good detection results (better than random) while the space below the diagonal line represents poor ED (worse than random). Hence, the ED will be less accurate if the curve is closer to the 45-degree diagonal of the ROC space (Figure 4). The closer the curve follows the left-hand border and the top border of the ROC space, the more accurate the ED process will be (Figure 4). The area under the curve is also a measure of the detection accuracy. A larger area under the curve means that there is better ED accuracy and vice versa (Figure 4). The ROC concept, as a frequently used concept for evaluating the PU signal detection efficiency, is further used in the results section of this work.

## 5. Energy Detection Process

### 5.1. Detection and False Alarm Probabilities

According to (5) and (6), the detection and the false alarm probabilities can be expressed as statistical probabilities in order to become [45,47]:

(11) $$P_{d_{i}}=Prob\;\left(\tau_{i}\left(n\right)>\lambda_{i}\left(n\right)\right)=Q\left(\frac{\lambda_{d_{i}}-\left(P+\sigma_{n_{i}}^{2}\right)}{\sqrt{\frac{2}{N}}\left(P+\sigma_{n_{i}}^{2}\right)}\right)$$

(12) $$P_{fa_{i}}=Prob\;\left(\tau_{i}\left(n\right)<\lambda_{i}\left(n\right)\right)=Q\left(\frac{\lambda_{fa_{i}}-\sigma_{n_{i}}^{2}}{\sqrt{\frac{2}{N}}\sigma_{n_{i}}^{2}}\right)$$

where Q(.) is the standard Gaussian complementary cumulative distribution function (CDF). Based on relations (11) and (12) and by taking into account that $\lambda_{fa_{i}}=\lambda_{d_{i}}=\lambda_{i}$*,* the interdependence between the detection probability and the false alarm probability can be developed. For the ED process not impacted by NU and performed with a fixed detection threshold, this interdependence can be expressed as:

(13) $$P_{d_{i}}=Q\left(\frac{Q^{-1}\left(P_{fa_{i}}\right)-\sqrt{\frac{N}{2}}\;SNR}{1+SNR}\right)$$

where $Q^{-1}\;\left(.\right)$ is the inverse standard Gaussian complementary CDF. According to relation (13), for a small sample quantity, low SNR and high false alarm probability, the PU signal detection cannot be achieved for any level of detection threshold $\lambda_{i}$.

### 5.2. Detection Threshold Estimation

The choice of detection threshold is the most important process that defines the performance of any sensing method, including the ED [8]. The detection threshold is a value that defines the detection efficiency, and optimal threshold selection represents the value needed to meet the detection performance requirements [3]. As previously mentioned, the threshold estimation techniques can be broadly classified as either fixed or dynamic. The fixed threshold can be calculated based on two principles: the constant false alarm rate (CFAR) and the constant detection rate (CDR). Noise power ($\sigma_{n_{i}}^{2}$) is needed to determine the threshold in both cases [3,49].

If the required false alarm probability ($P_{fa_{i}}$) is predetermined, the false alarm threshold based on the CFAR principle ($\lambda_{fai}$) can be expressed according to relation (12) as [47,49]:

(14) $$\;\;\lambda_{fai}=Q^{-1}\;\left(P_{fa_{i}}\right)\sigma_{n_{i}}^{2}\sqrt{\frac{2}{N}}+\sigma_{n_{i}}^{2}$$

In the CFAR principle, the threshold is set to meet an aimed false alarm probability ($P_{fa_{i}}$) and this threshold is then used to calculate the corresponding detection probability $P_{d_{i}}$. The CFAR principle is used in CR when it is required to guarantee the reuse probability of the unused spectrum that demands the setting of the probability of the false alarm $P_{fa_{i}}$ to a small fixed value, while the detection probability $P_{d_{i}}$ should be maximised.

Similarly, in order to achieve a target detection probability $P_{d_{i}}$ for the average received signal power *P* of a specific PU, the detection probability threshold $\lambda_{d_{i}}$*,* in the case of the CDR principle, can be derived from relation (11) [47,49]:

(15) $$\lambda_{di}=Q^{-1}\left(P_{d_{i}}\right)\left(P+\sigma_{n_{i}}^{2}\right)\sqrt{\frac{2}{N}}+\left(P+\sigma_{n_{i}}^{2}\right)$$

The CDR principle is used when it is necessary to guarantee a non-interference probability of the incumbent systems. This requires setting the detection probability $P_{d_{i}}$ to a high value and minimising the false alarm probability $P_{fa_{i}}$. By comparing expressions (14) and (15), it can be noted that the CFAR approach does not need the average received signal power *P* of a PU to set the threshold $\lambda_{fa_{i}}$. For this reason, the CFAR principle is more commonly used in practice.

However, constantly setting the false alarm probability $P_{fa_{i}}$ to low values means that the corresponding threshold ($\lambda_{fai}$*)* will be high. As a consequence, interference may occur since it is not easy to detect low-power signals due to the very demanding values of the CFAR threshold $\lambda_{fa_{i}}$. Therefore, the CFAR approach based on a fixed threshold is not optimal. An optimal threshold setting can be achieved if each SU dynamically adjusts its threshold according to its channel state.

In order to gain better system performance, selecting the appropriate value of the DT is a challenging task. However, performing analyses when DT variation is taken into account enables significantly more realistic analyses. Instead of remaining constant, the threshold can be scaled with DT factor $\rho^{\prime }$ ($\rho^{\prime }\geq 1$*)* in such a way that the factor $\rho^{\prime }$ sets the DT interval $\lambda_{i}^{\prime DT}\epsilon \left[\frac{\lambda_{i}}{\rho^{\prime }},\;\rho^{\prime }\lambda_{i}\right]$.

In the case of an ED system based on DT adaptation ($\lambda_{i}^{\prime DT}$), the detection probability (from relation (11)) and the false alarm probability (from relation (12)) are given by:

(16) PdiDT=minλi′DTϵ[λiρ′,  ρ′λi]Q(λi′DT−(P+σni2)2NDT(P+σni2))=Q(λiρ′−(P+σni2)2NDT(P+σni2))

(17) PfaiDT=maxλi′DTϵ[λiρ′,   ρ′λi]Q(λi′DT−σni22NDTσni2)=Q(ρ′λi−σni22NDT σni2)

The interdependence between the detection probability and the false alarm probability can be expressed as:

(18) $$P_{d_{i}}^{DT}=Q\left(\frac{Q^{-1}\left(P_{fa_{i}}^{DT}\right)-\left[\rho^{\prime 2}SNR+\left({\rho^{\prime }}^{2}-1\right)\right]\sqrt{\frac{N^{DT}}{2}}}{{\rho^{\prime }}^{2}\left(1+SNR\right)}\right)$$

If the detection threshold is fixed, the factor  ρ ′=1, and relations (16), (17) and (18) converge to (11), (12) and (13), respectively. The case when factor  ρ ′>1 implies that the ED process is based on the adaptation of the DTs. A higher value of the DT factor results in a greater range of DTs for possible selection.

### 5.3. Noise Uncertainty Estimation

The threshold values of (14) and (15) are derived based on the knowledge of the exact noise variance $\sigma_{n_{i}}^{2}$. However, in practice, it is very difficult to assume the exact level of noise variance in any moment. This is a consequence of the fact that the total noise can vary significantly from time to time since it consists of the noise from the receiver and environmental and thermal noise together. This noise power fluctuation, known as NU, causes a decrease in the accuracy of the sensing sensitivity. The drawback of this phenomenon is that the accuracy of detection falls quickly, which can cause SU interference in the PU [45]. Therefore, neglecting the existence of NU leads to a limitation in assessing the performance of the ED.

To achieve a more realistic scenario, it is necessary to take into account the impact of the NU variations on the ED process. Uncertainty of noise power can be expressed by the NU factor *ρ* (*ρ* ≥ 1). Therefore, the bounds of the noise variance ($\sigma_{NU_{i}}^{2}$) are assumed to be in the interval σNUi2ϵ[σni2ρ, ρσni2] defined by NU factor ρ. The detection probability and the false alarm probability in the case of NU can be derived from (11) and (12), respectively. The expressions for detection probability and the false alarm probability are:

(19) PdiNU=minσNUi2ϵ[σni2ρ,ρσni2]Q(λi−(P+σNUi2)2NNU(P+σNUi2))=Q(λi−(P+σni2ρ)2NNU(P+σni2ρ))

(20) PfaiNU=maxσNUi2ϵ[σni2ρ,ρσni2]Q(λi−σNUi22NNU σNUi2)=Q(λi−ρ σni22NNU ρ σni2)

According to relations (19) and (20), the probability of PU detection when the received signal is impacted by NU and the fixed detection threshold is:

(21) $$P_{d_{i}}^{NU}=Q\left(\frac{\rho Q^{-1}\left(P_{fa_{i}}^{NU}\right)-\left(SNR\;-\;\frac{\rho -1}{\;\rho }\right)\sqrt{\frac{N^{NU}}{2}}}{\frac{1}{\rho }+SNR}\right)$$

The selection of factor ρ=1 implies that there is no NU and relations (19), (20) and (21) converge towards relations (11), (12) and (13), respectively. However, the case when ρ>1 implies the existence of NU and the larger values of *ρ* mean a larger NU range. For example, when the NU factor is 1.02, it means that the variations of the noise power are 2% of the average level of received noise power.

### 5.4. Energy Detection with NU and DT Adaptation

The ED system that jointly encompasses DT adaptation and the estimation of uncertainty in the noise level (NU) is the most realistic from the implementation and simulation perspective. However, it is also the most difficult to implement in practice because it requires the highest processor power in terms of the SU device. This is due to the necessity of the continuous estimation of noise variance (NU) and DT during the sensing process.

In this section, the PU signal detection probability and the false alarm probability will be expressed as functions of NU and DT. Hence the limits of NU variation are assumed to be in the interval σNUDTi2ϵ[σni2ρ, ρσni2] while the limits of the DT adaptation are assumed to be in the interval $\lambda_{i}^{\prime NUDT}\epsilon \left[\frac{\lambda_{i}}{\;\rho \;\prime },{\;\rho \;\prime \;\lambda }_{i}\right]$. By taking into account these limiting values, the false alarm probability and the detection probability in the case of NU and ED performed by DT adaptation can be derived based on the central limit theorem, thus becoming [44]:

(22) PdiNUDT=minλi′NUDTϵ[λiρ′  ρ′λi]  minσNUDTi2ϵ[σni2ρ,   ρσni2] Q(λi′NUDT−(P+σNUDTi2)2NNUDT(P+σNUDTi2))=Q(λiρ′ −(P+σni2ρ)2NNUDT (P+σni2ρ))

(23) PfaiNUDT=maxλi′NUDTϵ[λiρ′,ρ′λi]maxσNUDTi2ϵ[σni2ρ,ρσni2]Q(λi′NUDT−σNUDTi22NNUDTσNUDTi2)==Q(ρ′λi−ρσni22NNUDTρσni2)

It is possible to define the number of samples needed or sensing duration period as a function of $P_{d_{i}}^{NUDT}$, $P_{fa_{i}}^{NUDT}$ and average SNR as [47]:

(24) $$N^{NUDT}=\frac{2[{\left(\rho /\rho^{\prime })Q^{-1}\left(P_{fa_{i}}^{NUDT}\right)-\rho^{\prime }\left(1/\rho +SNR\right)Q^{-1}\left(P_{d_{i}}^{NUDT}\right)\right]}^{2}}{{\left[\rho^{\prime }SNR+\rho^{\prime }/\rho \;-\rho /\rho^{\prime }\right]}^{2}}$$

The $N^{NUDT}$ represents the minimum sample quantity needed for the precise detection of PU signal for specified NU factor ρ and DT factor $\rho^{\prime }$. It can be observed that the relation (24) does not contain the decision threshold parameter. This means that, for any threshold level, the detection with a higher sample quantity must result in a better probability of ED, and vice versa. According to relations (22) and (23), the detection probability ($P_{d_{i}}^{NUDT}$) and the false alarm probability ($P_{fa_{i}}^{NUDT}$) are related as [47]:

(25) $$P_{d_{i}}^{NUDT}=Q\left[\frac{\frac{\rho }{\rho^{\prime }}Q^{-1}\left(P_{fa_{i}}^{NUDT}\right)-\left(\rho^{\prime }SNR+\frac{\rho^{\prime }}{\rho }-\frac{\rho }{\rho^{\prime }}\right)\sqrt{\frac{N^{NUDT}}{2}}}{\rho^{\prime }\;\left(SNR+\frac{1}{\rho }\right)}\right]$$

To target the specific PU signal detection probability, relation (25) shows that parameters, such as the average SNR, the probability of a false alarm $P_{fa_{i}}^{NUDT}$ and the sampling number $N^{NUDT}$, must be guaranteed for specific factors of DT adaptations ($\rho^{\prime }$) and NU variations (ρ).

**Figure 5:**
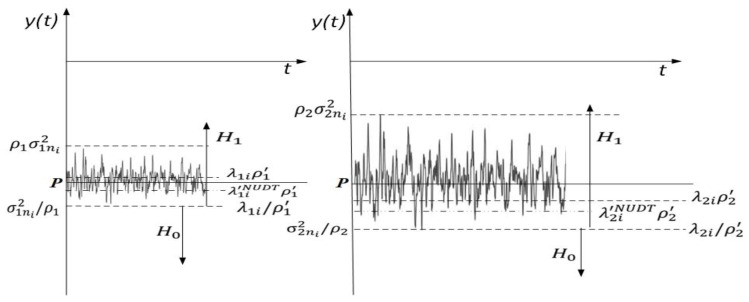
DT and NU ranges in the ED process of signals with equal average received power.

The influence of NU and DT (as separate factors) on overall ED performance can be simulated based on three cases by selecting appropriate values of $\rho^{\prime }$ and ρ. For the first case, where $\rho^{\prime }$ = 1.00 and ρ > 1.00, there is no continuous adaptation of DT. In addition, the second case, where $\rho^{\prime }$ > 1.00 and *ρ* = 1.00, means that the ED process excludes the impact of NU and the only DT adaptation is simulated in the ED process for sensing PU signals. For the same channel characteristics (e.g., the same SNR at the location of SU) impacted by NU and the same sample quantity $N^{NUDT}=N^{NU}$, the detection probability and false alarm probability, expressed with relations (22) and (23), converge to relations (19) and (20) for the first case and relations (16) and (17) for the second case. In the third case, the influence of NU and DT adaptation on the ED process can be simulated when ρ > 1.00 and $\rho^{\prime }$ > 1.00. In order to illustrate the impact of these specific factors, Figure 5 visualises two signals with equal average received powers (|*P*| = $\int_{0}^{t}y\left(t\right)$*dt*) but different NUs (variations). The received signal presented on the left side in Figure 5 has a lower noise variance, which is in the range $\sigma_{1NUDT}^{2}{}_{i}\epsilon \left[\frac{\sigma_{1n}^{2}{}_{i}}{\rho_{1}},\;\rho_{1}\sigma_{1n}^{2}{}_{i}\right]$, while the received signal presented on the right side has significantly higher noise variance spanning the range $\sigma_{2NUDT}^{2}{}_{i}\epsilon \left[\sigma_{2n}^{2}{}_{i}/\rho_{2},\;\rho_{2}\;\sigma_{2n}^{2}{}_{i}\right]$, where $\rho \prime_{2}>\rho \prime_{1}$. Hypotheses $H_{0}$ or $H_{1}$ from relations (1) or (9–10) are satisfied if the signals from Figure 5 fall below or above the dynamically set threshold, respectively. The detection threshold is dynamically adjusted according to the signal level and noise variations (NUs), and it can be in the range λ1i′NUDTϵ[λ1iρ′1, λ1iρ′1] for the left side signal in Figure 5 and λ2i′ NUDTϵ[λ2iρ′2, λ2i ρ′2] for the right side signal where ${\rho \prime }_{2}>{\rho \prime }_{1}$. In essence, the detection threshold has to be set based on the worst-case noise level uncertainty. In order to maximise the detection probability, the threshold value has to be adjusted appropriately. This means that an increase in NU must be followed by an appropriate change in DT adaptation (Figure 5).

## 6. Energy Detection Algorithm

The algorithm developed for simulating ED of OFDM signals is presented in this section. The algorithm enables the simulation of the detection process for PU signals with and without DT adaptation and NU variations. Matlab (R2016) was used to model the SS process and to simulate the ED of the signals transmitted within different OFDM system designs (RA, MA and combined RA and MA).

The pseudocode of the algorithm used for the SS of signals based on the ED method is presented as Algorithm 1. The 1st line of Algorithm 1 sets the input simulation parameters, which include the OFDM signal (*ofdm_signal*), the sample quantity (*N)*, the length of the OFDM data (*len_ofdm_data)* obtained after conversion of signals from parallel to serial (as shown in Figure 1), the SNR, the NU factor (ρ), the DT factor (ρ′), the noise variances $\left(\sigma_{n_{i}}^{2}\right)$, the range of $P_{fa_{i}}$ values *(length (*$P_{fa_{i}}$*))* and the number of Monte Carlo simulations *(kk)*. Table 3 shows the exact values of the parameters used in the simulation. The selected values are based on the parameters characteristic of the real OFDM systems.

**Algorithm 1** Simulation of ED process*1:INPUT:OFDM signal (ofdm_signal), len_ofdm_data, sample quantity (N), SNR, NU factor (ρ), DT factor (*ρ′*), noise variance (*$\sigma_{n_{i}}^{2}$*), length of (*$P_{fa_{i}}$*), and number of Monte Carlo simulations (kk)* *2: OUTPUT: Detection probability (*$P_{d_{i}}^{NUDT}$*)* *3: INITIALISE: OFDM signal (ofdm_signal)* *Step 1: Simulation Detection probability (*$P_{d_{i}}$*) vs. Probability of False Alarm (*$P_{fa_{i}}$*) based on (13, 18, 21, 25)* *4:       set kk = number of Monte Carlo simulations* *5:       set Pfa = false alarm probability in interval [0,1]* *6:  FOR   p = 1:length (*$P_{fa_{i}}$*);* *7:      i1 = 0; i2 = 0;* *8:  FOR   kk = 1:10,000;* *Step 2: Generate AWGN noise (*$w_{i}(t)$*) with zero mean and variance* *9:         Noise_1 (ρ = 1.00,*ρ′*> 1.00) = sqrt (*$\sigma_{n_{i}}^{2}=1.00$). *randn (1, len_ofdm_data); *10:       Noise_2 (ρ > 1.00,*ρ′*> 1.00) = sqrt (*$\sigma_{n_{i}}^{2}>1.00$. *randn (1, len_ofdm_data); *Step 3: Generate PU signal*$x_{i}$*(t) and Received signal*$y_{i}$*(t) calculation* 11:    final_ofdm_signal = sqrt(SNR).*ofdm_signal; *12:    received_signal_1 = final_ofdm_signal + Noise_1;* *13:    received_signal_2 = final_ofdm_signal + Noise_2;* *Step 4: Received signal energy calculation* *14:    energy_calc_1 = abs(received_signal_1).^2;* *15:    energy_calc_2 = abs(received_signal_2).^2;* *Step 5: Test statistic calculation using (2)* 16:    test_stat_1 = (1/N).*sum(energy_calc_1); 17:    test_stat_2 = (1/N).*sum(energy_calc_2); *Step 6: Threshold evaluation using (17) and (23)* *18:              thresh1(p) = ((qfuncinv(*$P_{fa_{i}}$*(p))./sqrt(N))+ 1)./*ρ′*;* 19:              thresh2(p) = ((qfuncinv($P_{fa_{i}}$(p)).* ρ./sqrt(N))+ ρ)./ρ′*;* *Step 7: Decision making using (7) and (8)* *20:            **IF** (test_stat_1 >= thresh1(p));* *21:            i1 = i1+1;* *22:           **END*** *23:           **IF** (test_stat_2 >= thresh2(p));* *24:                 i2 = i2 + 1;* *25:           **END*** *26:   **END*** *Step 8: Monte Carlo simulation-determining*$P_{d_{i}}$ (*1)* *27:* $P_{d_{i}}$
*1(p) = i1/kk;* *28:* $P_{d_{i}}$
*2(p) = i2/kk;* *29:**END*** *30:**UNTIL***
$P_{d_{i}}$
*= [0,1]*

The OFDM signal (*ofdm_signal)* in line 3 of Algorithm 1 is generated by setting the initial parameters, which include the OFDM modulation type, the size of each OFDM block, the FFT/IFFT points, the length of CP, the interference constellation and the normalisation type and amount of PU Tx power. The parameters for performing the Monte Carlo simulations, such as the length of the false alarm probability ($P_{fa_{i}})$ and the number of simulations, are defined and executed in Algorithm 1 lines 4 to 8. Lines 9 to 10 show the pseudocode expressing generation of AWGN with variance $\sigma_{n_{i}}^{2}$ and zero mean. The selected values of noise variances used for the simulation are set to realistic levels characteristic for real wireless channels (Table 3).

The OFDM signal (*final_ofdm_signal*) is created by multiplying the amount of SNR and the values of the OFDM signal in line 11. In lines 12–13 of Algorithm 1, two versions of the received signal are shown. The *Received_signal_1* indicates the OFDM signal without the noise variation (ρ=1) detected with the DT adaptation ($\rho^{\prime }>1$). The *Received_signal_2* indicates the OFDM signal in the scenario of NU variation (ρ>1) detected with DT adaptation ($\rho^{\prime }>1$). The energy calculation for each of the received signals (*energy_calc_1 and energy_calc_2*) is presented in lines 14–15 of Algorithm 1.

In pseudocode lines 16–17, the average signal received for a number of *N* samples is represented as a calculation of test statistics for two scenarios: the test statistics for signals received with DT adaptation (*test_stat_1)* and for signals received with NU variation and DT adaptation (*test_stat_2)*. The calculation of the test statistics expressed as average received signal energy is performed according to the relation (2).

Pseudocode lines 18–19 present the assessment of the received signal threshold. *Thresh1(p)* expresses the first scenario where the received signal is detected by means of DT adaptation. *Thresh2(p)* expresses the second scenario where the received signal is impacted by the NU variation and received based on DT adaptation. Mathematical expressions for the first and second scenario are presented by relations (17) and (23), respectively.

The decision process is performed according to the relations (7) and (8) and presented in lines 20–26 of Algorithm 1. For every scenario analysed, the comparison of the threshold is performed using the corresponding received signal energy: *test_stat_1* and *test_stat_2*. If the received signal energy is equal to or larger than the threshold, hypothesis $H_{0}$ is confirmed and PU is present as indicated in relation (1). If the received signal energy is lower than the threshold, hypothesis $H_{1}$ is confirmed (according to relation (1)) and PU is absent. In lines 27–30 of the algorithm, determining the probability of PU signal detection ($P_{d_{i}}$) is performed through Monte Carlo simulations in order to obtain the most realistic results for all cases analysed. The false alarm probability ($P_{fa_{i}})$ passes through a set of values in the range 0–1. For each false alarm probability ($P_{fa_{i}})$, the detection probability ($P_{d_{i}}$) is calculated in order to be in the range 0–1. Analyses are performed for the different values of DT $\left(\rho^{\prime }\right)$ and NU (ρ) factors, which differ among the specific simulation cases.

## 7. Results of Simulations

This section presents the parameters used in simulations and cognitions obtained as a result. The ED approach as an SS method was simulated for the diverse OFDM based systems and ED concepts (with and without DT adaptations and NU variations). Differences between the received PU signals (in terms of Tx power, modulation types, SNR levels) and ED approaches (in terms of sample quantity, target false alarm probabilities) were simulated through the impact of NU variations and DT adaptations on the ED process. Based on the simulations performed, a presentation of the impact of these factors on the ED capabilities of different OFDM system designs is given.

### 7.1. Parameters of Simulation

Table 3 shows a summary of the parameters used in the simulations. The four most common types of OFDM transmission modulations were used: 256 QAM, 64 QAM, 16 QAM, and QPSK. As indicated in Table 3, the FFT sizes of OFDM signals equal to 1024, 512, 256 and 128 were selected for analysis. The SNR of the received signals selected for analysis was between −25 dB and 10 dB. Such an SNR range covers the practical environments of many contemporary communication technologies based on OFDM transmission. The signal detection and false alarm probabilities were analysed for the range 0–1. The results were obtained for 10,000 Monte Carlo simulations (Table 3). This number of Monte Carlo simulations was selected for analysis according to the trade-off between simulation duration and simulation accuracy. For the modeling of the different types of received PU signals, the characteristic values of the NU and DT factors were used (Table 3). To eliminate the influence of NU (*ρ*) and DT (*ρ′*) in the modeling, the NU and DT factors were set to ρ *=* ρ′ = 1.00. To simulate a more realistic PU signal detection, NU and DT factors in the range between 1.01 and 1.05 were exploited for modeling of the different impacts of the NUs and DTs on the ED process (Table 3). The analysis was based on the worst-case detection and false alarm probabilities when the NU and DT are in the ranges specified by the ρ and ρ′ parameters.

### 7.2. Influence of DT Adaptation on Performance of ED

**Figure 6:**
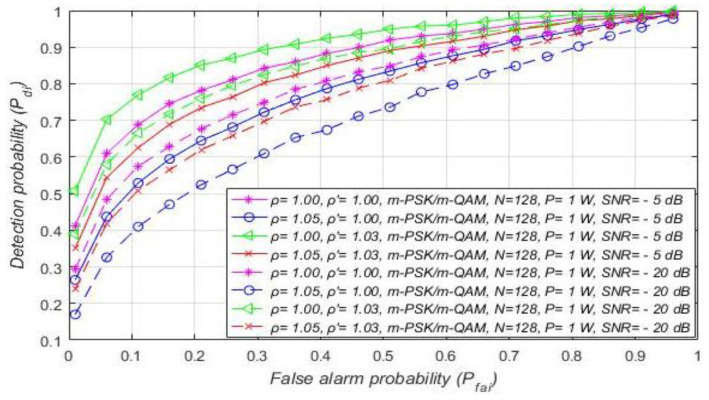
Interdependence between probabilities of detection and false alarm for ED of signals in RA systems, versatile combinations of NU and DT factors, and two distinct SNR levels.

The results of the first study presented in Figure 6 show the influence of the DT adaptation on the ED process in RA systems. The results were obtained for a constant Tx power (1 W) of PU, and distinct levels of DT ( ρ ′) and NU factors (ρ). Different NU factors express the influence of the intensity of AWGN variations on the ED of OFDM signals. Additionally, different DT factors represent a versatile level of threshold adaptation capabilities during the ED process.

Figure 6 presents the results obtained as ROC curves for the distinct constellations of m-PSK/m-QAM modulations at the location of SU. The results presented in Figure 6 were also obtained for two SNR values (−5 dB, −20 dB) and the unchangeable number of OFDM samples (*N* = 128). These conditions are typical of real, practical implementations in which ED performance is challenged by different SNRs in the position of SU. The obtained results presented in Figure 6 show that the detection probability ($P_{d_{i}})$ is equal for any m-QAM or m-PSK modulation. Hence for any such systems transmitting with a constant Tx power (RA systems), the detection probability does not depend on the modulation order. This is a consequence of the nature of the PU transmission in the case of RA systems, which is based on the dynamic adjustment of the modulation order while transmitting at the same Tx power. For this reason, the signal energy detected at the location of the SU during the ED in RA systems can only be affected by noise fluctuation. This supports the results presented in Figure 6 showing that, for the same sample quantity, SNR in the position of SU, and the Tx power of PU, the detection probability will be lower for higher noise variations (higher NU factor ρ).

**Figure 7:**
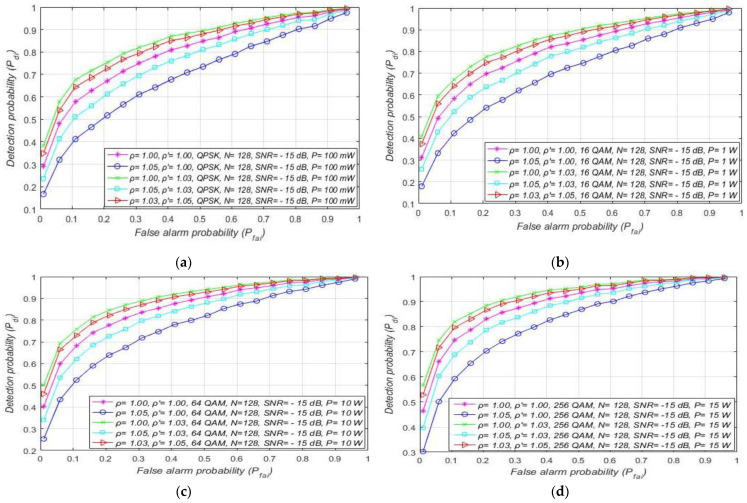
Interdependence between detection and false alarm probabilities for ED of signals in combined RA and MA system under versatile combination of NU and DT factors and PU transmission using: (**a**) QPSK modulation at Tx power of 0.1 W; (**b**) 16 QAM modulation at Tx power of 1 W; (**c**) 64 QAM modulation at Tx power of 10 W and (**d**) 256 QAM modulation at Tx power of 15 W.

Although in RA systems the adjustment of modulation order during ED does not have any effect on the PU signal detection probability, it can be seen from Figure 6 that DT adjustment can improve ED performance. More specifically, in the case of ED without NU variations and with DT adaptation ($\rho =1.00,\;\rho^{\prime }=1.03$), the detection probability will be the highest for any modulation scheme (Figure 6). However, this scenario is, from a practical point of view, the least realistic. According to Figure 6, the lowest detection probability was obtained for the simulation scenario with significant noise variations (ρ=1.05), but without DT adaptation ($\rho^{\prime }=1.00)$. This is expected since the ED of signals impacted by NU and without DT adaptation during the ED process significantly reduces the detection probability. Hence, the improvement of the detection probability in the case of increased NU variations for all modulation schemes (Figure 7) can be expected only if ED is based on DT adaptation ($\rho^{\prime }>1$).

In Figure 7, the results of the analyses dedicated to the influence of DT adaptation on ED of PU signals in combined MA and RA systems are presented. The results were obtained for a fixed SNR (min. −15 dB) at the location of SU, a constant sample quantity (*N* = 128), and for the four most commonly used OFDM modulations: QPSK and 16/64/256-QAM. The simulation results are presented in Figure 7 as ROC curves for distinct NU levels and DT factors, and the different PU Tx powers and corresponding modulations. The levels of the Tx power selected for the analyses are characteristic of real OFDM communication systems, such as WLAN (100 mW) and 2G–5G wireless mobile systems (1 W for the mobile device, 10–15 W for macro base stations).

Figure 6 and Figure 7 suggest that, for a specific SNR at the location of SU and for ED of signals with an equal sample quantity *N* impacted by an equal NU variation (ρ), the detection probability will be higher in the cases where PU transmits with a larger Tx power and vice versa. This is a consequence of a lower Tx power, which results in lower energy at the location of SU that further results in a lower signal detection probability. The results presented in Figure 7 also show that the larger DT adaptation range (higher DT factor $\rho^{\prime })$ results in a better detection probability and, consequently, the better performance of the ED process. It is shown that, for the larger range of NU fluctuations (a higher NU factor ρ), better ED performance can be accomplished only if the larger range of DT adaptations are used. This enables the appropriate adjustment of the threshold to the larger NU variations. However, an uncontrolled setting of the DT adaptation range to some arbitrary high or low value can lead to the selection of too low or too high ED thresholds. This might lead to extreme ED sensitivity or misdetection (Figure 5). In both cases, the detection probability will be degraded. Hence the DT range must be adapted according to the level of the NU variations. This means that the higher expected NU variation should be followed by a higher DT adaptation range and vice versa.

**Figure 8:**
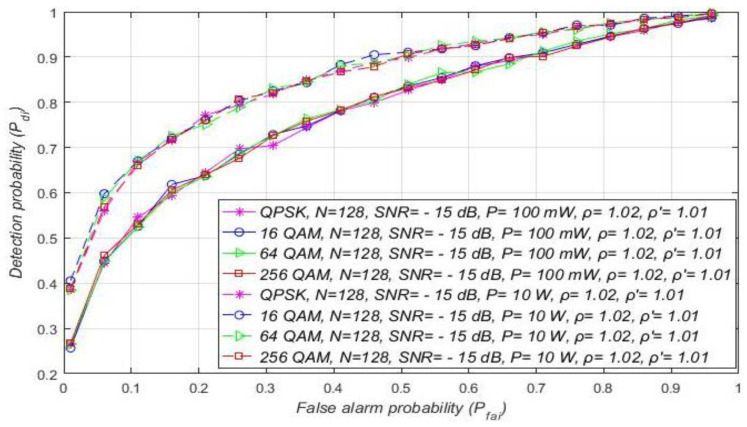
Interdependence between detection and false alarm probability for ED of signals in MA systems transmitted with two Tx powers and four different OFDM modulations.

Additional analyses have been performed for the MA systems affected by the same NU variation (ρ = 1.02). Figure 8 presents the results of the analyses, where it was assumed that every OFDM modulation (QPSK or 16/64/256 QAM) for a specific level of Tx power (10 W or 100 mW) can obtain SNR equal to −15 dB or higher at the location of SU. The simulation results present distinctions among the detection probabilities for MA systems transmitting with versatile Tx powers, while using the same OFDM modulation.

The higher detection probability will involve the sensing of the OFDM modulated signals transmitted at a higher Tx power for the same false alarm probability. This leads to the conclusion that the Tx power adaptation in MA systems largely influence the detection probability, even when the NU variation is constant (Figure 8).

### 7.3. Impact of SNR on the ED Performance

**Figure 9:**
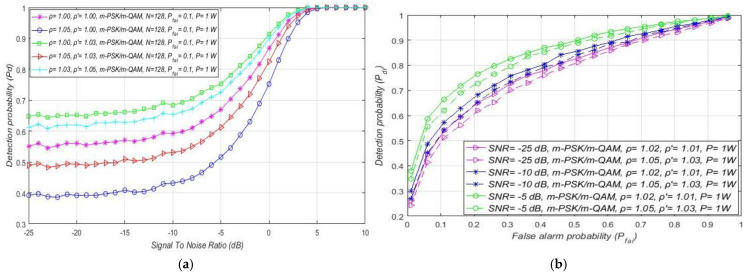
(**a**) Detection probability vs. SNR for signals transmitted in RA systems; (**b**) Interdependence between probabilities of detection and false alarm for versatile NUs, DTs and SNRs in the case of RA system.

Next, simulations were performed with respect to the influence of SNR at the location of SU on the ED performance. The obtained results shown in Figure 9a for the RA systems indicate the effect of SNR on the detection probability of the OFDM signal. The results presented in Figure 9a confirm that, for the constant PU Tx power equal to 1 W and for the unchanged value of the false alarm probability equal to $P_{fa_{i}}$ = 10%, the detection probability will remain the same for any modulation and corresponding constellation (m-QAM//m-PSK). Nevertheless, Figure 9a shows that for the higher levels of SNR, the detection probability will be better for any modulation order and decrease with the degradation of SNR. This is due to the higher SNR, which results in higher PU signal energy at the location of SU. This improves the ED process and, as a consequence, increases the PU detection probability. In addition, the results presented in Figure 9a confirm that the trade-off between the level of NU variation and DT adaptation has an impact on the detection probability. More specifically, for the higher NU variation range (ρ = 1.05) and lower DT adaptation range *(*$\rho^{\prime }=1.03$), the detection probability will be lower compared to the ED of the signals impacted with the lower NU variation (ρ = 1.03) and ED performed with a higher DT adaptation ($\rho^{\prime }=1.05$).

Additionally, the ROC curves for the fixed PU Tx power (1 W) and the versatile SNR values (−5 dB, −10 dB, −25 dB) of OFDM signal influenced with distinct NUs and received with different DT adaptation ranges are presented in Figure 9b. The presented results show that, for channels with a higher SNR in the location of SU (−5 dB), the detection probability will be higher due to the lower influence of noise on the ED. Although NU variations have an impact on the ED, the total AWGN level has a significant influence on the ED and high levels of noise can largely reduce ED efficiency. This is due to the lower SNR in the position of SU, where the fixed PU Tx power means higher noise. As a consequence, this results in a lower detection probability. Apart from the significant influence of the overall level of AWGN, Figure 9b additionally confirms the non-negligible influence of NU, which further contributes to the degradation of ED efficiency. Figure 9b suggests that, for lower NUs, the detection probability will be higher for signals with a higher SNR. Based on these results, the combination of high SNR with low NU positively influences the ED performance of the RA system.

**Figure 10:**
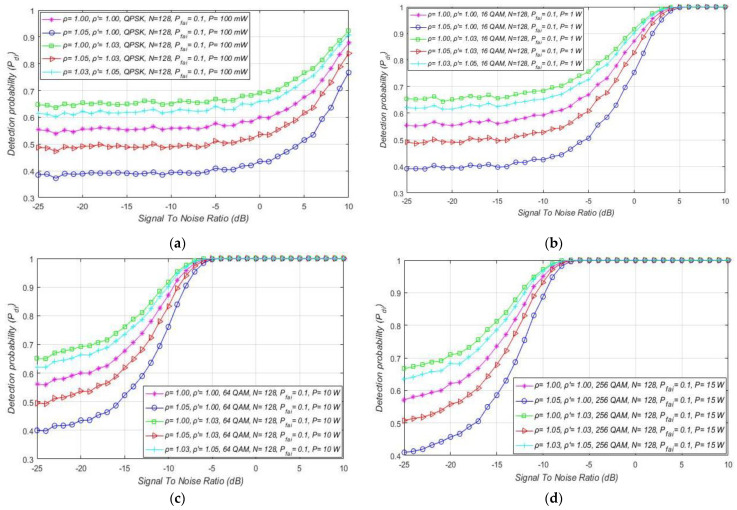
Impact of SNR on the detection probability of OFDM signals received under versatile combinations of NU and DT factors when PU transmits at: (**a**) 0.1 W; (**b**) 1 W; (**c**) 10 W and (**d**) 15 W.

For combined MA and RA systems, the impact of SNR on the detection probability of the OFDM signals affected by distinct NUs is presented in Figure 10a–d. The results show that, independent of the OFDM modulation and PU Tx power, larger noise fluctuations (characterised with a larger NU factor ρ) decrease the detection probability at any SNR lower than the SNR threshold (confirmed PU detection). The fluctuations of NU influence every OFDM signal, regardless of its modulation order and Tx power. Although for the signals with a lower Tx power this influence is more observable, this is also reflected in the lower detection probabilities (Figure 10a–d).

Furthermore, for the distinct levels of Tx power, an SNR threshold higher than some level for which the PU detection probability can be ensured is visible (Figure 10a–d). This SNR threshold is significantly affected by the Tx power level and is reduced for the signals with larger PU Tx (e.g., 5 dB for Tx power of 1 W and −5 dB for 10 W Tx power). According to Figure 10a–d, the OFDM constellation has no influence on this SNR threshold, since it has no direct impact on the detection probability (which is also confirmed with relations (11), (13), (16), (18), (19), (21), (22) and (24)).

**Figure 11:**
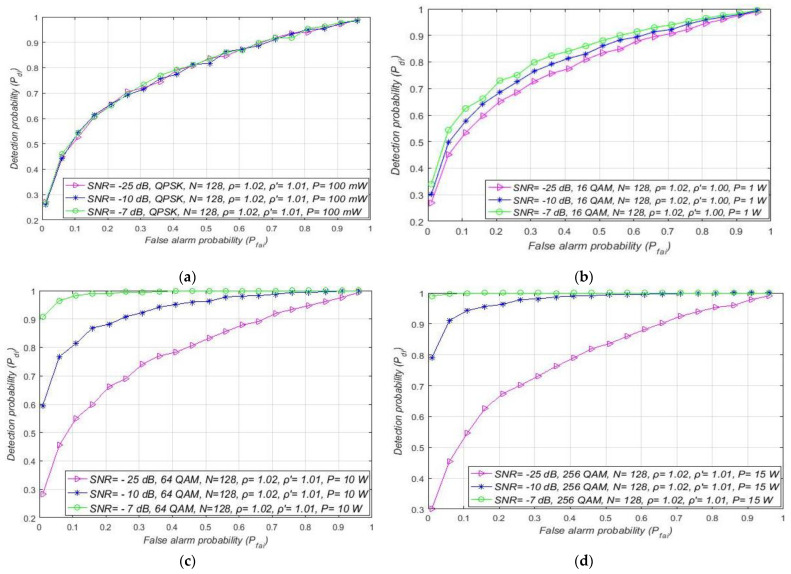
Interdependence between detection and false alarm probability for ED of m-PSK/m-QAM modulated signals received under different SNR levels when PU transmits at: (**a**) 0.1 W; (**b**) 1 W; (**c**) 10 W and (**d**) 15 W.

The impact of distinct SNR levels and Tx powers on the detection probability of combined MA and RA systems is shown in Figure 11. The results were acquired for distinct SNRs (−7 dB/−10 dB/−25 dB) of the m-PSK/m-QAM signals influenced by the same NU (ρ = 1.02) in the scenario where the PU signal is being transmitted at different Tx powers (15 W, 10 W, 1 W and 0.1 W). Based on the results obtained, the combination of the level of PU Tx power and SNR at the position of SU has a strong influence on the detection probability for the ED technique. According to expectations, for better SNR and higher Tx power levels, the detection probability will be larger and vice versa. Moreover, in Figure 11a, it can be seen that, for the low Tx power values equal to 100 mW, the detection probability at the location of SU cannot be increased without SNR enlargement (above −7 dB). Otherwise, in Figure 11d, it can be seen that, for the larger Tx power equal to 15 W, the signal detection during ED can be ensured (detection probability of $P_{d_{i}}$= 100%) for each SNR level higher than −7 dB. These prove the strong influence of the trade-off between the noise and Tx power on the efficiency of ED.

**Figure 12:**
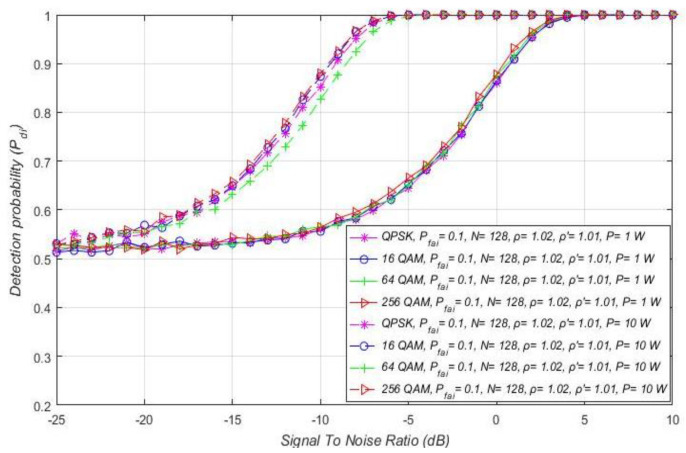
Detection probability vs. SNR for MA system with PU transmitting at distinct Tx powers (1 W/10 W) using four different OFDM modulations.

Figure 12 shows the impact of SNR on the detection probability of the MA systems, which transmit at distinct Tx powers equal to 1 W/10 W. Transmission is assumed to occur over a channel with equivalent channel environments (the same NU factor ρ = 1.01), the fixed false alarm probability ($P_{fa_{i}}$ = 10%), and versatile OFDM modulations. If the modulated OFDM signals are transmitted with the equal Tx power and influenced by the same NU, the results presented in Figure 12 indicate that the OFDM modulations do not have any influence on the detection probability at any SNR level. Nevertheless, a lower detection probability can be seen for signals of equivalent OFDM constellations transmitted at a lower Tx power. This is because transmission at a lower Tx power degrades the SNR in the position of SU. For higher SNRs in the position of SU (e.g., above −5 dB), the MA system that transmits with a lower Tx power (1 W) will have a lower detection probability than an MA system transmitting at a larger Tx power having the same SNR in the position of SU. Therefore, in MA systems, transmission at a lower Tx power degrades the detection probability at any SNR in the position of SU. However, transmission at a lower Tx power has a positive impact on the power consumption of the PU device.

### 7.4. Impact of the Sample Quantity on the ED Performance

**Figure 13:**
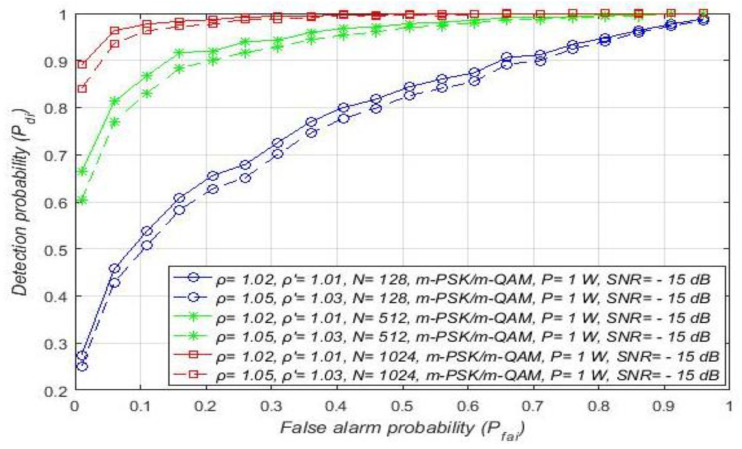
Interdependence between detection and false alarm probabilities for ED of m-PSK/m-QAM modulated signals transmitted with Tx power of 1 W and sensed with the distinct sample quantities.

The following results presented in this section are dedicated to the analysis of the influence of the sample quantity (*N*) on the capability of detecting PU during the ED process. The ROC curves for signals transmitted with a constant Tx power equal to 1 W using the m-QAM/m-PSK modulations and detected by a different sample quantity are presented in Figure 13. Analyses were performed for two combinations of NU and DT factors (ρ = 1.02, $\rho^{\prime }=1.01$ and ρ =1.05, $\rho^{\prime }=1.03$) and for the case when SNR is fixed in the position of SU equal to −15 dB.

Figure 13 shows that the sample quantity has a non-negligible influence on the ED performance of RA systems. When the smaller sample quantity is used in the ED process (*N* = 128), the results obtained for the channels with the same channel characteristics (equivalent NU and SNR) showed that the signal detection probability will be lower and vice versa. This result is a consequence of the lower sample quantity, which actually means a lower number of independent trials in a period during which the signal of PU is being sensed.

**Figure 14:**
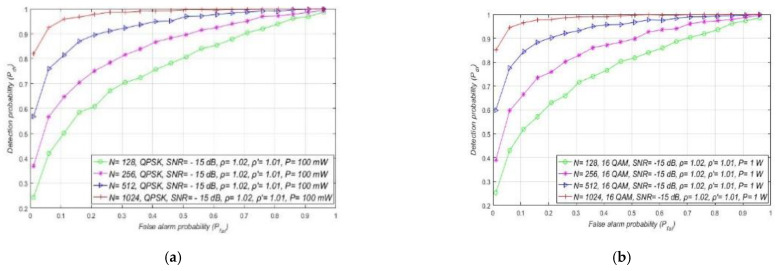

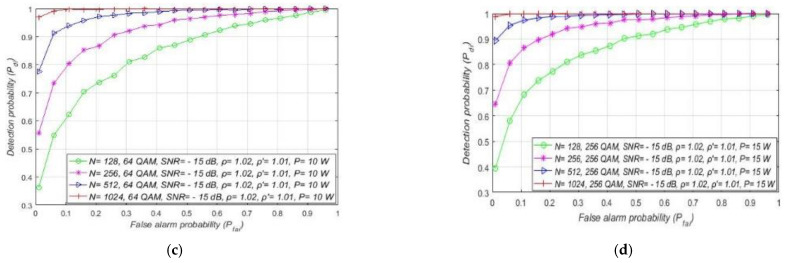
Interdependence between detection and false alarm probabilities for ED of versatile OFDM signals detected with different sample quantities and for PU transmission at Tx power: (**a**) 0.1 W; (**b**) 1 W; (**c**) 10 W and (**d**) 15 W.

Compared to the detection of signals impacted by a higher NU and somewhat higher DT factor (ρ = 1.05,  ρ ′=1.03), the results in Figure 13 show that, for the lower values of NU and DT factors *(*ρ = 1.02,  ρ ′=1.01), a higher detection probability can be obtained with the same sample quantity. This is because the lower NU results in less degradation of the received signal, which demands less sample quantity (sensing attempts) for the accurate ED of the PU signal. To ensure the same probabilities of detection for the same sample quantity, a significant increase of NU must also be followed by an appropriate increase in the DT adaptation. Otherwise, the sample quantity must be increased and this trade-off between sample quantity and DT adaptation range significantly impacts the ED efficiency. ROC curves have been shown in Figure 14a–d for different OFDM modulated signals sensed by a distinct sample quantity and transmitted at four distinct Tx powers. The analysis was performed for the fixed SNR in the position of SU equal to −15 dB and for moderate NU level (ρ= 1.02) with the corresponding DT adaptation ( ρ ′=1.01).

Based on the results presented, the increase in the sample quantity and PU Tx power in combined MA and RA systems led to an increase in the detection probability and vice versa. This was an expected result since relations (11), (16) and (19) confirm that the sample quantity (*N*) and the Tx power of PU influence the detection probability. A lower Tx power for some OFDM modulations means weaker signal energy at the location of SU. Furthermore, fewer sample quantity *N* results in a lower number of independent trials for the PU ED.

Additionally, for analysed combinations of NU, Tx power, and SNR level (higher than the SNR wall) presented in Figure 14d, a threshold *N,* related to the sample quantity above which the detection probability can be ensured for each OFDM modulation ($P_{d_{i}}$ = 100%), can be seen. For OFDM modulations transmitted at a higher Tx power (presented in Figure 14), the value of this threshold will be lower. Transmission at larger Tx power means more energy at the location of the SU user which requires fewer samples to perform accurate PU detection. Since higher Tx power is generally used for transmission of modulations with a lower constellation order and vice versa, in combined MA and RA system designs this type of transmission can obtain PU detection with a smaller sample quantity.

**Figure 15:**
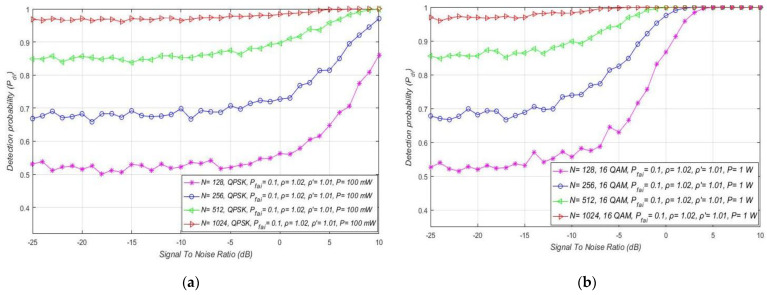

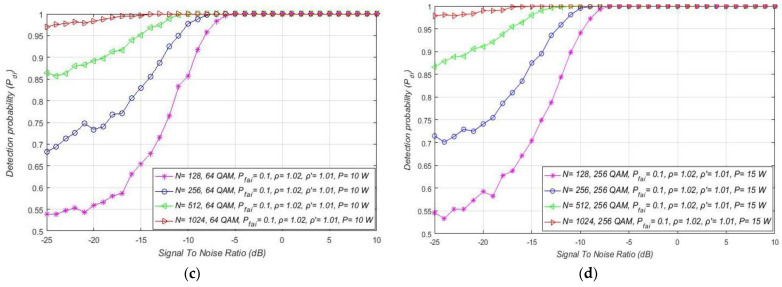
Influence of SNR and sample quantity on the detection probability in combined RA and MA systems transmitting at: (**a**) 0.1 W; (**b**) 1 W; (**c**) 10 W and (**d**) 15 W.

The impact of the sample quantity on the detection probability ($P_{d_{i}}$) for MA systems is shown in Figure 15a–d. The results were obtained for the fixed false alarm probability ($P_{fa_{i}}$ = 0.1), the DT factor  ρ ′=0.1, and transmission at four distinct Tx powers (100 mW, 1 W, 10 W, 15 W). Analyses were also performed for channels with a constant NU variation equivalent to 2% of the average noise level. The results obtained showed that a lower detection probability is accomplished for a lower SNR, and a lower sample quantity for any OFDM modulation (Figure 15a–d). Since a lower SNR is a direct result of the impact of higher noise on signal transmission at a specific Tx power, the detection probability will decrease as the level of SNR decreases (Figure 15a–d).

However, Figure 15a–d indicate the presence of the SNR threshold below which the detection probability cannot be guaranteed ($P_{d_{i}}\;$< 100%). The value of the threshold will be larger for signals sampled with a larger sample quantity or transmitted with a higher Tx power. Therefore, increasing the detection probability is related to the trade-off between the Tx power of the PU in MA systems and the sample quantity used during ED by SU. According to this trade-off, for a higher Tx power and lower sample quantity, it is possible to obtain better detection probabilities when compared to the PU transmission of signals with a low Tx power detected using an increased sample quantity.

This confirms the importance of the PU Tx power level in the ED performance of any OFDM based system (MA, RA, or combined MA and RA).

### 7.5. Influence of the False Alarm Probability on the ED Performance

The last group of simulation results is dedicated to the explanation of the impact of the false alarm probability on the detection probability for versatile OFDM-based systems. The false alarm probability ($P_{fa_{i}}$) is defined (in Section 4.1.) as the probability that the SU wrongly concludes that a PU is transmitting when the PU is not active. In the case that a real PU is present and the SU correctly estimates this, the possibility that the SU wrongly concludes that a PU is present starts to increase. This explains why the decrease in the false alarm probability ($P_{fa_{i}}$) is followed by a decrease in the detection probability ($P_{d_{i}}$) and vice versa (indicated in Figure 6, Figure 7 and Figure 8, Figure 9b, Figure 11, Figure 13 and Figure 14).

**Figure 16:**
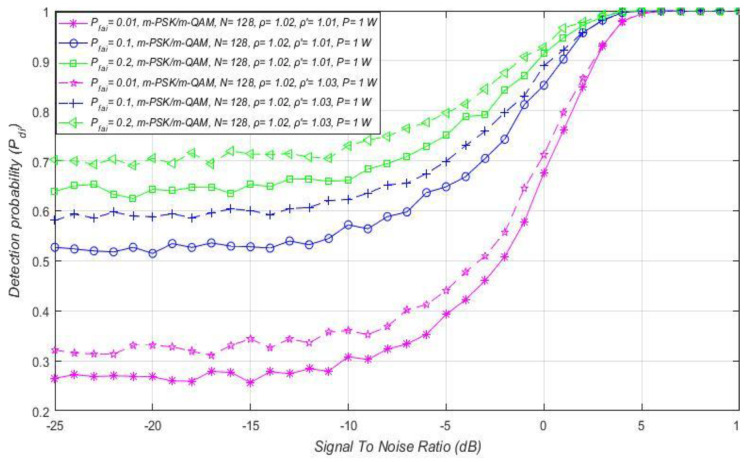
Detection probability vs SNR relationship for RA system with distinct false alarm probabilities and different combinations of NU and DT factors.

As mentioned in Section 5.2., performing the ED process based on satisfying the demand for some predefined value of the false alarm probability is the essence of the CFAR ED process. Although the false alarm probability must be as low as possible, in practice a false alarm probability of up to 20% can be tolerated and used. This motivates the selection of such a value in further analyses. In particular, the results of the analysis presented in Figure 16 were obtained for the three characteristic probabilities of false alarms ($P_{fa_{i}}$). From the practical point of view, they express low (1%), moderate (10%) and substantial (20%) false alarm probabilities. In Figure 16, for the RA system, the relationship among the detection probability and SNR has been presented with respect to the three values of false alarm probability. The results were obtained for an equal NU factor (ρ = 1.02), equal sample quantity (*N* = 128), and different DT factors ( ρ ′=1.01, ρ ′=1.03).

The obtained results showed that, for lower values of SNRs and probabilities of false alarm (1%), the detection probability will be lower and vice versa (Figure 16).

As explained in Section 7.2., a low SNR results in a decrease in detection probability (Figure 6, Figure 7 and Figure 8, Figure 9b, Figure 11, Figure 13 and Figure 14). However, for ED with a low level of PU signal energy in the position of SU, and for a decrease in the false alarm probability to the lowest values, a decrease in detection probability will be obtained (Figure 6, Figure 7 and Figure 8, Figure 9b, Figure 11, Figure 13 and Figure 14). However, for ED with a higher DT factor ( ρ ′=1.03), Figure 16 shows a higher detection probability for the same false alarm probability, SNR, and signals impacted by the same NU. This further proves the importance of DT adaptations, which can improve the ED process for any value of demanded false alarm probability in the case of the CFAR ED approach.

**Figure 17:**
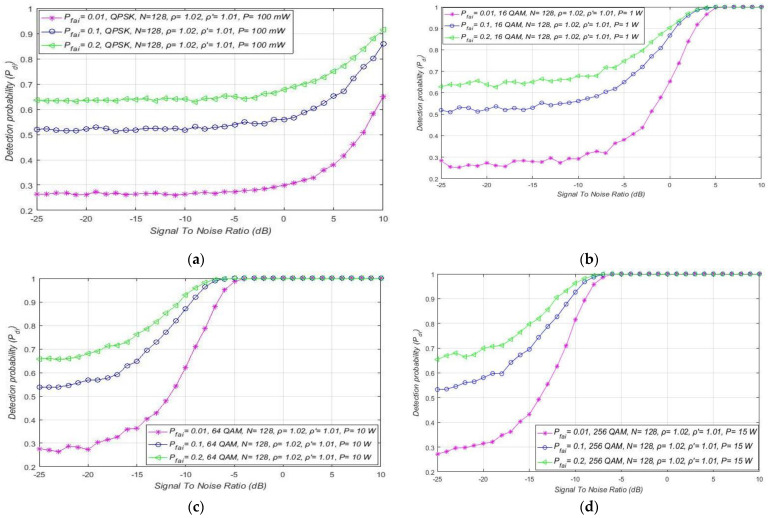
Detection probability vs. SNR relationship for distinct false alarm probabilities in combined MA and RA systems with PU transmitting at: (**a**) 0.1 W; (**b**) 1 W; (**c**) 10 W and (**d**) 15 W.

The impact of different SNRs on detection probability for combined MA and RA systems transmitting at distinct Tx powers is presented in Figure 17a–d. The results for each of the four simulations were obtained under equal NU variation (ρ = 1.02), the same sample quantity (*N* = 128), and DT factor ( ρ ′=1.01). In Figure 17a–d, it can be seen that the combination of a higher Tx power and a higher false alarm probability increases the detection probability for any OFDM constellation. Nevertheless, it must be taken into account that the increase in false alarm probability raises the possibility of incorrect decisions by the SU in the ED process. For this reason, an appropriate trade-off between Tx power and maximal false alarm probability in case of the CFAR ED approach must take place. Additionally, Figure 17a–d show that the SNR threshold (known as the SNR wall) below which accurate PU signal detection is not feasible is influenced by the false alarm probability and PU Tx power. According to Figure 17a–d, the SNR threshold will be lower for signals transmitted with a larger Tx power (−5 dBm for Tx power of 100 mW compared to −25 dB for Tx power of 15 W) and detected by the lower (CFAR) setting of false alarm probability. For this reason, in combined MA and RA systems, OFDM signals transmitted at a higher PU Tx power can be sensed at lower SNRs, if the set value of the false alarm probability is higher.

## 8. Future Challenges for the Enhancement of ED Limitations

The discussion concerning the performance limitations and future research opportunities for the improvement of the ED method is given in this section. The results of simulations shown in the preceding section on the ED effectiveness of versatile OFDM based systems were used as the foundation of this discussion. Table 4 provides an overview of the main disadvantages of the ED method and points out the opportunities for the enhancement of SS based on ED.

### 8.1. Research Challenges Related to Noise Uncertainty

The results of the simulations shown in Figure 6, Figure 7, Figure 9, Figure 10 and Figure 13 confirm that the major ED weakness is the reduction of the detection precision when the variation of NU (noise power) rises. As shown in the preceding section, the degradation of the ED performance caused by the NU can be lowered through the DT adaptation (Table 4).

Thus far, the literature has presented significant research results dedicated to the topic of DT adaptation for the improvement of the ED technique [3,8,10,16,45,47,50,51,52,53,54,55,56,57,58,59,60,61,62,63,64]. To satisfy the requirement of the targeted probability of a false alarm, in Refs. [51,60,63,64], the detection threshold was optimised iteratively. In Ref. [51], an optimal adaptive threshold that utilises the SS error function was proposed. In Ref. [53], the SS based on unknown noise power was adaptively estimated for the different methods of ED. For finding and localising the narrowband signals, in Refs. [5,54,130], an algorithm based on double-thresholding was proposed. Generally, references [50,51,52,53] showed the degradation of the sensing performance when the energy detector does not properly adjust its threshold. A well-selected detection threshold can minimise the SS errors, ensure the appropriate detection of a PU, and enable full spectrum utilisation [3].

Several authors have investigated the implementation of DT when there is an NU influence on the sensing process in CR networks. In Refs. [55,56,57,58], the SS performance of ED was presented by considering the NU and different DTs in the channel. Different types of DT methods, more specifically Otsu, Rosin, Kapur and Entropy, were presented and compared in Ref. [15]. The impact on the ED sensing performance of NU and DT as both separate and joint factors was analysed in Ref. [36]. Besides their ratio, it was concluded that the absolute values of DT and NU have a significant impact on sensing performance. In Ref. [45], DTs were arbitrarily generated for each SU in order to increase the detection performance of the predefined NUs.

An ED method based on an adaptive threshold, which keeps the false alarm rate at a preferred point under any noise level in an unknown WGNC, was presented in Ref. [59]. The adaptation of the SS threshold in CR by means of a method based on discrete Fourier transform filter bank was proposed in Ref. [60]. In Ref. [8], the adaptive threshold method was proposed as an alternative approach to estimate the threshold as a function of the first and second-order statistics of recorded signals. The simulation results indicate that the adaptive threshold has a low false alarm rate when the standard deviation coefficient of the noise is selected properly. This approach can satisfy the detection requirements of multi-channel CRs for either narrow or wideband SS.

The adaptive threshold control for ED was implemented in Refs. [60,63] with a linear adaption of the threshold based on SINR (Signal to Interference plus Noise Ratio) [61]. This approach achieves a much higher SU throughput than the energy detectors with a fixed threshold, while maintaining the good stability of false alarm and missed detection probabilities. The proposed approach is based on the concept of estimating channel noise in which only noise is received by the SU. In Ref. [62], the usage of DT computed based on minimising the probability of error while keeping the transmission rate was proposed. The result indicated that the probability of an error occurring can be minimal when the DT scheme is used, while the noise variance can be constant or variable in the channel. In Ref. [49], an estimated noise variance was used to calculate the DT for the SS based on ED.

In Ref. [10], a new adaptive threshold algorithm was proposed for the discrete wavelet packet transform and Welch’s ED methods to assess the trade-off between detection and false alarm probability. The proposed adaptive algorithm demonstrated that the target performance requirements can be achieved for very low SNRs (−18 dB).

According to the results presented, the constant change in noise power over time makes the dynamic ED threshold adaptation a demanding activity and no optimal algorithm has been deduced so far. Thus, the enhancements in the algorithms which will set the iteratively optimal DT in any moment of ED process remain an open research issue for SS based on the ED method. Previous related works have mostly analysed the adaptation of the DT through simulations with different system parameters assumed to be unchanged in simulations. Since parameters can change over time, the assessment of new algorithms for ED must be performed in practice.

### 8.2. Research Challenges Related to the ED Degradation at Low SNRs

ED performance significantly depends on an estimate of the noise level in terms of precision and reliability. Since this estimate is used for calculating SNR at the position of SU, the evaluation of the influence of different DT adaptation levels for versatile NU levels was presented in the previous section. According to the results presented in Figure 9, Figure 10, Figure 11 and Figure 12 and Figure 15, Figure 16 and Figure 17, an additional relevant disadvantage of the ED method is the lack of possibility to reliably sense PU signals for low SNRs at the location of SU (Table 4), which was also proven in Refs. [66,67].

According to the results of the simulations presented previously, if the noise power contributes to the overall SNR in such a way that the SNR is below a specific level known as the SNR wall [68], the energy detector cannot sense a difference between the PU signal and the somewhat higher noise power. This works independently of the sensing duration length or the used sample quantity [55]. Since measuring NU is a demanding process due to the constant variations of NU over time, ED techniques require a mechanism that constantly estimates NU in order to accurately compute the overall noise power.

This estimation can be done using OFDM signal guard bands or by means of a channelized radiometer in the frequency domain [69,70]. The channelized radiometer divides the overall frequency spectrum into several channels and then combines the energy from each separate channel using a radiometer. The cell averaging concept presented in Ref. [68] was used for the realisation of channelized radiometer when CFAR ED strategies are considered [70]. An estimation concept based on an adaptive noise level was offered in Ref. [67], as a consequence of the errors detected in the assessment of noise power [56]. This concept was based on classification algorithms of multiple signals, which can split the noise and signal subspaces and estimate the noise floor. An approach based on the DT selection according to the real measurements of the noise level power present in the received signal during SS was proposed in Ref. [65]. The results showed an enlargement in the detection probability compared to those based on ED with a static threshold.

Nevertheless, new approaches offering more reliable and accurate noise level estimation for SS using ED are missing. Such novel approaches which will increase the precision of the noise level estimation can contribute to the improvement of signal detection at weaker SNRs (Table 4).

### 8.3. Research Challenges Related to Sensing Duration

The next important ED limitation is detection accuracy reduction when performing detection with a low sample quantity (Table 4). This reduction is also confirmed by the simulation results presented in Figure 13, Figure 14 and Figure 15. ED requires a large sample quantity and/or a longer sensing period if a high detection probability is to be accomplished [72,73,131]. However, during the sensing period, the end-to-end transmission delay increases since the data transmission is stopped, which also degrades the SU throughput. For these reasons, the sample quantity used for the ED should be as low as possible. This has a negative impact on the detection performance (as presented in Section 7.4) and requires more repetitions of the sensing period. Hence, the sensing time (sample quantity) and periodic sensing intervals in the CR networks must be optimised to minimise sensing errors and/or to increase the SU throughput (Table 4). The strong impact on the ability of the SU to exploit the available spectrum involves an adjustment of the periodic sensing interval during the ED process [66,74,75].

Moreover, it was shown in Section 7.3 that the sample quantity impacts the ED accuracy in terms of the detection probability. The trade-off between the SU throughput and sensing duration of the ED method was analysed in Ref. [76]. In order to optimise the transmission rate for the Sus, under the limitation that the PUs are adequately secured, the sample quantity used in the detection process is minimised. The authors in Ref. [16] proposed a DT adaptation to improve the detection performance in an environment characterised by NU and low SNR. The results showed a considerable reduction in the minimal number of samples for sensing at low SNR compared to other proposed approaches in the literature. Nevertheless, the frequency of the sensing period (in terms of the sample quantity) and its duration is an important design element that requests further investigation for the improvement of ED SS (Table 4).

To address the problem of weak detection precision in the case of detection with a low sample quantity, solutions based on SS with a dual-radio sensing architecture have been proposed [81,82]. With the dual-radio detector approach, one radio chain is dedicated to SS, while the other is dedicated to data reception and transmission. Compared to the single-radio architecture, the possible drawbacks of such an approach are the increased hardware cost and power consumption of SU.

An ED shortage related to the impairment of detection accuracy in case of a small sample quantity is further dedicated to the ability of using the ED in cognitive wireless sensor networks (CWSN). ED can be very promising for use in CWSN, since sensor nodes characterised with low power consumption can exploit the low computational complexity of the ED method [77,78,79]. However, increasing the sample quantity for precise sensing reduces the energy efficiency of the sensor nodes. In CSWN, even maintaining the transceiver of the sensor node active only during SS contributes to large power consumption [80]. Hence, the possible application of the ED method in resource-limited CWSN demanding maximisation of the sensors’ battery lifetime can only be realised through the development of novel ED concepts (Table 4). These concepts must combine a minimum duration of sensing and a low computational complexity of the ED process.

### 8.4. Research Challenges Related to the Different SS Methods

Among different narrowband methods for local SS, the ED is just one of the non-cooperative methods (Table 4). To improve the sensibility and reliability of the available spectrum detection, thus far in the relevant literature a number of distinct narrowband SS approaches have been proposed. The comparison of the main performance parameters among ED and other relevant non-cooperative local SS methods is presented in Table 5.

Regarding the accuracy of detection at different SNRs, the ED method is less accurate compared to the cyclostationary feature detection (CFD), entropy detection (END), goodness of fit test detection (GFTD) and eigenvalue based detection (EBD) methods (Table 5). Additionally, for the challenging case of the detection of signals at low SNR, matched filter detection (MFD) and waveform based detection (WBD) have significantly better detection accuracy than ED (Table 5). In the case of WBD and MDF methods, this is due to the necessity of obtaining a priori knowledge of some of the PU signal parameters. Therefore, the amount of information received from PU needed for performing accurate SS significantly differs among the local SS methods compared in Table 5. While END, GFD and EBD do not require any a priori information about the PU signal, the aforementioned MFD, WBD and CFD techniques request precise information about the PU signal pattern. This information might include details such as the transmitter pilots, preamble, guard bands, etc., while WBD even requests the synchronisation between the PU and SU. However, accurate prior information about the PU signal may not be achievable at any time, since SU and PU do not exchange information constantly. This represents the main limitation of the MFD, CFD, and WBD techniques.

Nevertheless, for the MFD and WBD methods, it has been shown that the accurate prior information of the PU signal contributes to a reduction of sensing frequency and has an equal or even better detection performance for lower sample quantity when compared to other local sensing methods (Table 5). Compared with the ED method, the CFD, END and EBD methods request a similar sample quantity for accurate SS. This is significantly larger than for the MFD and WBD methods when equal detection accuracy has to be accomplished. This is an important disadvantage which limits the practical implementation of these methods in CWSNs, since a higher sample quantity and more frequent sampling periods contribute to the faster depletion of wireless sensor batteries. Compared with the ED method, the MFD, END, WBD and EBD methods are more robust against NU while the GFTD technique has a similar level of robustness (Table 5). However, CFD is considerably more resistant against NU than ED and the other methods, due to the fact that the noise is typically not cyclostationary. The more accurate PU signal detection in the channels impacted by the higher NU variation can be accomplished by exploiting the cyclostationarity effect of the PU signal in the case of the CFD method. In the case of the EBD method, more accurate PU detection is observed for better correlation structure and diversities in the eigenvalues of the statistical covariance matrix of noise and signal.

However, in order to accomplish this in practice, the computational complexity of the CFD and EBD methods is significantly higher than the ED and other SS methods (Table 5). This was confirmed in the tutorial work [136] dedicated to the analysis of blind SS (BSS) approaches (requesting no prior knowledge of the PU signal), where a comparison between ED and the EBD covariance approaches was performed. Generally, when compared with other BSS or non-BSS methods (Table 5), the ED technique has the least amount of computational complexity since it does not encompass complex processing of signals. The low computational complexity of the ED method enables the simple implementation of ED and explains why ED compared with other local (non-cooperative) methods for SS currently has the highest representation in real life implementation.

The presented comparison of distinct local methods for SS shows that ED has a number of weaknesses (Table 5), and there is no optimal non-cooperative SS technique for every application. Choosing the most appropriate SS technique for local PU signal detection is a major challenge because SS techniques differ in their performance (Table 5). The implementation of an appropriate detection technique is strongly dependent on the application and it must be PU system-oriented in order to maximise the probability of detecting spectral opportunity.

These cognitions bring up the question of whether it would be possible to improve sensing accuracy through the parallel implementation of different local SS techniques at the same SU node (Table 4). Such cooperative SS can be of particular interest for the purpose of wideband SS, where, in the first stage, a low complexity detection technique (such as ED) can be used to search for possible idle sub-bands. Additionally, in the second stage, more advanced SS techniques (such as MFD, CFD, END) with higher detection sensitivity can be used whenever the desired performance or accurate idle band detection must be achieved. Some works have proposed a two-stage SS approach with a simple detection method used in the first stage and a more accurate and computationally complex method in the second stage [135,136,137,138]. A side effect of such an approach is found in the increased sensing time and SU hardware costs. This explains why cooperative SS is far from full exploitation. Hence, significant research activities must take place to precisely address the role and advantages of ED as a method that can be used in conjunction with other local SS methods at the same SU.

### 8.5. Research Challenges Related to the Fading Channels and Hidden Node Problem

Although ED in low SNR environments cannot achieve accurate and reliable sensing results, previous research has also shown that ED, being a simple technique, lacks detection reliability in deep fading environments [66,67,124] (Table 4). The performance analysis of ED in fading channels was performed in Refs. [115,116,117,118,119,120,121]. Additionally, setting the detection threshold with respect to the frequency notches in wireless frequency-selective fading channels is one limitation. The other is the inability of the ED method to detect (direct sequence and frequency hopping) spread-spectrum signals [3]. The ED technique is also susceptible to spurious tones and baseband filter effects [137].

To overcome such challenges, more sophisticated SS methods based on collaboration among the SUs must be devised. Determining whether a collaboration between different SUs and corresponding SS techniques can improve the sensing performance in fading environments is a possible approach to overcoming some of the aforementioned limitations (Table 4). Collaborative detection is based on utilising versatile SS techniques by spatially dislocated SUs in order to achieve accurate and reliable detection decisions. It was demonstrated by Refs. [68,116,122] that a collaboration among distributed SUs that employ ED alleviates the effects of NU when the users are experiencing independent and identically distributed fading or shadowing.

Additionally, ED in other non-cooperative local spectrum sensing methods (presented in Table 5) is sensitive to a hidden node problem (Table 4). When a cognitive SU is at a far distance from the PU, the PU signal may be too weak for reliable detection. An advantage of collaborative SS can be the possibility of improving the sensing performance through mutual communication between the SUs [123]. The information on the signal of the PU can be relayed to the distant SU. This can be done after reliable local signal detection has been performed by the SU located near the PU. The challenges related to the integration of SUs which use ED as the method for local SS in such a relaying collaboration detection concept have to be investigated. Hence. further experimental investigations are needed in order to improve the sensing performance in fading and hidden node environments, based on collaboration among SUs which can use ED as one of the sensing techniques.

### 8.6. Research Challenges Related to the Users’ Distinction, Interference and Wideband Sensing

It is emphasised that ED is appropriate for random signal detection, since the ED method does not demand any prior knowledge of the primary signal. Unfortunately, ED is not an appropriate sensing method if the efficient spectral opportunity utilisation is conditioned. This is because the ED method cannot distinguish between the interference, SU, and PU signals (Table 4). As the ED method cannot identify the interference, adaptive signal processing used for cancelling the interferer at SUs cannot be implemented. Moreover, the spectrum policy related to the use of some of the frequency bands is limited to PUs. A cognitive user should treat noise and signal from other SUs differently. In order to achieve this, the collaboration between different detection techniques among SUs represents a promising approach (Table 4).

Previous research in this field has been dedicated to collaborative multistage SS [125,126,127], including the local SS stage, the transmission of the sensing results stage and the stage of information fusion for making detection-related decisions. For any type of collaborative detection that can be centralised or decentralised [3], the local SS conducted by each SU is performed independently, utilising the same or different detection algorithms, such as those presented in Table 5 (ED, MDF, CFD, END, WBD, GFTD, EBD). Since ED is a lightweight and simple method, as elaborated in the previous sections, some works have already used this method within collaborative systems to assess local SS performance [129,132]. In this case, each SU transmits the detected binary decision or energy signal to the destination node. This node can be the neighbouring SU in the case of decentralised collaboration detection, or it can be a fusion centre (FC) in the case of centralised collaboration detection. However, the selection of the sensing method has an impact on how the cognitive SUs cooperate with each other. Sensing methods are essential in collaborative detection systems because the sensing and processing of PU signals is highly dependent on the Sus’ cooperation. Hence, the role of ED as the most popular SS method requests deeper investigation, especially in terms of cooperation with other SS approaches in such collaborative systems.

The benefit of this cooperation is found in the achievable space diversity, which brings a diversity gain afforded by the sensing performed from multiple independent SUs. Even if some of the SUs fail to detect the signal of the PU, detection opportunities for other SUs remain. This increases the probability of PU detection. As the number of SUs involved in cooperative SS increases, so does sensing accuracy and reliability. However, the negative effect of this is the increased complexity of such a collaborative sensing system. The question is, can ED as a simple method contribute to the reduction of this complexity? The coordination of the sensing policies involved, occurring between the collaborating SUs and the sensing cycles, must also be synchronised. Hence, an important challenge for collaborative cognitive systems is the coordination of the sensing policy. This coordination must be performed for the selection of the sensing frequency range, sensing inception, and sensing duration.

Additionally, wideband frequency sensing ensures the identification of more frequency opportunities. In the case of non-collaborative cognitive systems, this can be realised at the expense of time and hardware cost [3]. The possibility of alleviating these costs can be accomplished by wideband SS, using the implementation of a concept based on the collaborative detection approach. An approach based on collaborative sensing was proposed in Ref. [128]. In this approach, SUs in parallel sense different frequency bands simultaneously and transfer their estimations to the FC. The low-complexity ED method can be suitable in collaborative sensing for wider searching of possible idle sub-bands. However, comprehensive research in this area should be performed in order to assess the inclusion of the ED method in such a collaborative sensing approach.

Sensing inception and sensing duration must be coordinated among the SUs in order to minimise the time duration needed for identifying spectral opportunities and to maximise transmission time in collaborative sensing systems. However, opportunistic and/or dynamic spectrum access with ED as one of the sensing methods is still rudimentary. Different economic, regulatory and technical challenges must be solved before the advantages of the ED method can be fully exploited in collaborative sensing systems.

## 9. Conclusions

Due to its low implementation cost and complexity, the ED method is assumed to be a suitable approach for the practical realisation of SS in the CR networks. The broad implementation of the OFDM technique in contemporary communication is based on RA, MA and combined RA and MA systems. Such systems establish new possibilities related to using the OFDM technique in CR applications. In this survey paper, the ED of OFDM signals influenced by NU and detected through the adaptation of DT in the CR networks was thoroughly analysed.

Firstly, an explanation of the ED concept according to the mathematical formulation and a description of the influence of NU on the ED of the OFDM signals performed without and with DT adaptation were given. Then, using the developed algorithm, the ED of the OFDM signals transmitted using distinct OFDM-based systems was simulated and extensive simulation results were presented.

A comprehensive analysis of the obtained simulation results provided fundamental explanations concerning the impact of transmit powers, the sample quantity used for PU signal detection, SNRs at the location of SUs, and the false alarm probability on the ED probability when distinct OFDM system designs are used. Through systematic analysis of the obtained results, the weak performance of ED as an SS method was confirmed for SS with a low sample quantity in environments with low SNRs and with detection performed without DT adaptations. Improvements in ED performance were detected when the level of DT adoptions followed the intensity of the NU variations for all OFDM system designs. The analyses also showed a significant influence of different OFDM system designs on ED performance, since the system designs based on the Tx power adjustments had a strong impact on ED performance. By contrast, the system designs based on the OFDM modulation constellation adjustments did not have any direct impact on ED performance.

Furthermore, the open issues and research challenges related to the improvements of main ED limitations were analysed in detail through a survey of the existing literature. The analysis was performed in terms of possible improvements of the ED method related to the reduction of NU impact; the improvement of ED probability in the case of low SNRs; the benefits obtained through cooperation with different SS methods; the minimisation of the impact of fading; hidden nodes; and interference and the possibility of distinguishing between different users through cooperative detection. Opportunities for future research and directions which can bring improvements concerning ED as a SS method have been elaborated on. It has been shown that further research efforts are needed in order to address the drawbacks of ED and to make it possible to exploit all of the benefits of the ED method as the most frequently used SS method in practice.

## Figures and Tables

**Table 1:** Overview of ED methods analysed in the literature and their corresponding merits.

Approach Analysed in the Literature	Description of the Analysed Approach	The Merit of the Analysed Approach
Only the ED approach with fixed detection threshold [7,18,24,27,34]	No influence related to the impact of NU and DT adaptation	The strong impact of NU and lack of DT adaptation significantly degrade the reliability of ED performance
The ED approach impacted by NU and without DT adaptation [12,25,26,28,30,31,32,36]	Analyses of ED performance taking into account the impact of the NU variations	Reduced detection probability of ED method due to impact of NU variation caused by thermal noise and interference from neighbor communication systems
The ED approach with DT adaptation and without the impact of NU variations [8,10,11,13,37]	Analyses of ED performance based on the selection of the appropriate value of the DT	Somewhat improved detection probability of ED method due to exploitation of DT adaptation but less realistic due to neglecting the impact of NU variations
The ED approach impacted by NU variations and performed using DT adaptation [9,16,17,38,39,40]	Analyses of ED performance based on the selection of the appropriate value of the DT impacted by the NU variations	The best detection probability of ED method and the most realistic approach for simulation and analyses

**Table 2:** Parameters with their corresponding descriptions.

Index	Description
$H_{0}$	Hypothesis which determines the absence of the PU signal
$H_{1}$	Hypothesis which determines the presence of the PU signal
$y_{i}\left(n\right)$	Averaged received signal for *i-th* SU and for *n-th* sample
$w_{i}$(*n)*	AWGN signal for *i-th* SU
$\sigma_{n_{i}}^{2}$	Variance of AWGN signal for *i-th* SU without NU variations and DT adaptation
$\sigma_{NUDT_{i}}^{2}$	AWGN variance (interval) for ED with NU variations and DT adaptation
$\sigma_{NU_{i}}^{2}$	AWGN variance (interval) for ED with NU variations
x(n)	Transmitted *i-th* signal from the PU
$\tau_{i}$	Energy test statistic signal level of the detected signal
$\lambda_{i}$	Detection threshold of *i-th* SU *for ED* without NU variations and DT adaptation
$\lambda_{di}$	Detection threshold based on CDR and without NU variations and DT adaptation
$\lambda_{fai}$	False alarm threshold based on CFAR and without NU variations and DT adaptation
$\lambda_{i}^{\prime DT}$	DT (interval) for ED without NU variation
$\lambda_{i}^{\prime NUDT}$	DT (interval) for ED with DT adaptation and NU variation
$P_{d_{i}}$	Detection probability for ED without NU and DT
$P_{fa_{i}}$	False alarm probability for ED without NU and DT
$P_{d_{i}}^{DT}$	Detection probability for ED with DT adaptation
$P_{fa_{i}}^{DT}$	False alarm probability for ED with DT adaptation
$P_{d_{i}}^{NU}$	Detection probability for ED with NU variation
$P_{fa_{i}}^{NU}$	False alarm probability for ED with NU variation
$P_{d_{i}}^{NUDT}$	Detection probability for ED with DT adaptation and NU variation
$P_{fa_{i}}^{NUDT}$	False alarm probability for ED with DT adaptation and NU variation
*ρ*	NU factor
$\rho^{\prime }$	DT factor
*Q*	Standard Gaussian complementary CDF
$Q^{-1}$	Inverse standard Gaussian Complementary CDF
P	Average received PU signal power at position of SU
*N*	Overall sample quantity for ED without DT adaptation and NU variation
$N^{DT}$	Overall sample quantity for ED with DT adaptation
$N^{NU}$	Overall sample quantity for ED with NU variation
$N^{NUDT}$	Overall sample quantity for ED with DT adaptation and NU variation

**Table 3:** Parameters used in simulations.

Parameters	Quantity
Modulation of PU signal	OFDM
Type of OFDM (constellation)	QPSK, 16 QAM, 64 QAM, 256 QAM
Type of channel noise	AWGN
Quantity *N* of samples (FFT size)	128, 256, 512, 1024
SNR range at SU position (dB)	−25–10
The range of detection and false alarm probabilities	0–1
Quantity of Monte Carlo iterations per simulation	10,000
$\mathrm{Noise}\;\mathrm{variance}\;\sigma_{n_{i}}^{2}$ in the case of DT (*ρ* = 1.00)	1.00
$\mathrm{Noise}\;\mathrm{variance}\;\sigma_{n_{i}}^{2}$ in the case of NU and DT (*ρ* > 1.00, $\rho^{\prime }$ > 1.00)	1.01
NU factor ρ	1.00, 1.02, 1.03, 1.05
DT factor ρ′	1.00, 1.01, 1.03, 1.05

**Table 4:** Comparison of main ED drawbacks and expected improvements.

Major ED Disadvantages	Challenges in Future ED Research	Possible Enhancements
Reduction of detection precision as a consequence of variations in noise power (NU) [3,8,10,16,45,47,49,50,51,52,53,54,55,56,57,58,59,60,61,62,63,64]	Implementation of new algorithms for optimal selection of DT and its assessment	Improvement of ED accuracy in environments impaired with noise fluctuations (NU)
Degradation of detection accuracy at low SNRs [55,56,65,66,67,68,69,70,71]	Development of new noise estimation techniques	Improvement of ED accuracy at lower SNRs
Reduction of detection accuracy for a low sample quantity [72,73,74,75,76,77,78,79,80,81,82]	Optimising detection time (sample quantity) for exact detection	Increase in throughput of SU, reduction in SU energy consumption (in CWSN)
The existence of more accurate local SS methods [7,77,83,84,85,86,87,88,89,90,91,92,93,94,95,96,97,98,99,100,101,102,103,104,105,106,107,108,109,110,111,112,113,114]	Combining the ED approach with other approaches for SS at the same SU node	Ensuring desired sensing performance or accurate detection of specific idle bands
Degradation of detection accuracy due to fading channels and hidden node problem [66,68,115,116,117,118,119,120,121,122,123,124]	Novel collaboration approaches among SUs for reducing the impact of fading or hidden node problem	Improvement of ED robustness in fading channels and elimination of any hidden node problems
Inability to differentiate between interference, PU and SU signals [118,125,126,127,128,129]	Collaboration among distributed SUs based on combining ED with other detection methods in collaborative sensing	Elimination of the problem of PU, SU and interference distinction, and contribution to the improvement of wideband sensing

**Table 5:** Comparison of main performance parameters among ED and other non-cooperative SS methods.

Parameters for Comparison with ED Method	Matched Filter Detection (MFD)	Cyclostationary Feature Detection (CFD)	Entropy Detection Method (END)	Waveform Based Detection (WBD)	Goodness of Fit Test Detection (GFTD)	Eigenvalue Based Detection (EBD)
	[7,83,84,85,86,129,132,133]	[77,84,86,89,90,91,92,93,104]	[85,94,95,96]	[77,87,88,134,135]	[77,105,106,107,108,109,110,111,112,113,114]	[77,97,98,99,100,101,102,103,104]
Detection accuracy at all SNRs compared to ED	Significantly better	Better	Better	Significantly better	Somewhat better	Better
Amount of prior PU information compared to ED	Significantly higher	Higher	Equal (no PU information)	Higher	Equal (no PU information)	Equal (no PU information)
Sensing time (sample quantity) for accurate	Lower	Similar	Similar	Lower	Lower	Similar
Robustness against NU compared to ED	More robust	Significantly more robust	More robust	More robust	Similar	More robust
Computational complexity compared to ED	More complex	Significantly complex	More complex	More complex	Somewhat complex	More complex

## Abbreviations

The following abbreviations are used in this manuscript:

ADC	Analogue to digital converter
AWGN	Additive white Gaussian noise
BER	Bit Error Rate
BPF	Band-pass filter
BPSK	Binary Phase Shift Keying
CDF	Cumulative distribution function
CDR	Constant detection rate
CFAR	Constant false alarm rate
CFD	Cyclostationary feature detection
CP	Cyclic prefix
CR	Cognitive radio
CRN	Cognitive radio networks
CWSN	Cognitive wireless sensor networks
DT	Dynamic threshold
EBD	Eigenvalue based detection
ED	Energy detection
END	Entropy detection
FFT	Fast Fourier transform
GFTD	Goodness of fit test detection
GI	Guard interval
IEEE	The Institute of Electrical and Electronics Engineers
IFFT	Inverse Fast Fourier Transform
IoT	Internet of Things
ISI	Inter-symbol interference
LTE	Long Term Evolution
LTE-A	Long Term Evolution-Advanced
MA	Margin adaptive
MFD	Matched filter detection
NU	Noise uncertainty
OFDM	Orthogonal frequency division multiplexing
OFDMA	Orthogonal frequency division multiplexing access
PDF	Probability density functions
PU	Primary user
QAM	Quadrature Amplitude Modulation
QoS	Quality of service
QPSK	Quadrature Phase-Shift Keying
RA	Rate adaptive
RF	Radio frequency
ROC	Receiver Operating Characteristic
SINR	Signal to Interference plus Noise Ratio
SNR	Signal-to-Noise Ratio
SS	Spectrum sensing
SU	Secondary user
WBD	Waveform based detection
WiMAX	Worldwide Interoperability for Microwave Access
WLAN	Wireless Local Area Network
WRAN	Wireless Regional Area Networks
5G network	5th generation network
